# Breast Cancer Progression and Its Theranostic Management via Folate-Directed Targeting of Glycoprotein Receptor

**DOI:** 10.3390/medsci13040275

**Published:** 2025-11-19

**Authors:** Koyeli Girigoswami, Agnishwar Girigoswami

**Affiliations:** 1 Medical Bionanotechnology Laboratory, Department of Obstetrics & Gynaecology, Centre for Global Health Research, Saveetha Medical College, Saveetha Institute of Medical and Technical Sciences (SIMATS), Thandalam, Chennai 602105, TN, India; 2 Medical Bionanotechnology, Faculty of Allied Health Sciences, Chettinad Hospital & Research Institute (CHRI), Chettinad Academy of Research and Education (CARE), Kelambakkam, Chennai 603103, TN, India

**Keywords:** passive diffusion, active targeting, drug delivery, therapy, overexpressed receptors, health care

## Abstract

Breast cancer continues to rank among the most common and complex cancers worldwide. A promising approach is the direct delivery of drugs to cancer cells via specially designed nanocarriers that can target specific receptors on their surface, like folate receptors. When combined with other therapies, these functionalized nanocarriers can increase the effectiveness of treatment by more precisely targeting cancer cells than traditional methods that rely on passive targeting. Folate receptors are glycoproteins with four isoforms, for which both laboratory and animal models have shown encouraging results in research. The numerous chemical methods for attaching folic acid (FA) and enhancing drug delivery in folic acid-modified nanocarriers for breast cancer are examined in this review. Additionally, it examines how these smart carriers combine chemotherapy with alternative therapies like photodynamic therapies and state-of-the-art theranostics. The review highlights how important it is to carry out comprehensive testing to ensure that these innovations can successfully move from the lab to real clinical settings, even though the potential is evident.

## 1. Introduction

With multiple subtypes and unique occurrence patterns, breast cancer is a complicated and diverse disease. It accounts for roughly one-third of all female cancer cases globally and accounts for 15% of female cancer-related deaths [1]. Breast cancer develops and spreads around the world due to a combination of lifestyle, environmental, and genetic factors. Wealthier nations typically have lower death rates because of easier access to screening and treatment, but they also tend to see more cases, probably because of better detection. On the other hand, the number of cases is increasing in developing nations, primarily as a result of population growth and changes toward more Westernized lifestyles [2,3,4]. Globally, the leading cause of disability-adjusted life years (DALYs) is cardiovascular disease, followed by cancer. It is interesting to note that although high SDI (Sustainable Development Index) countries account for roughly half of all cancer cases, they bear only 25% of the total burden of cancer-related illnesses [5]. This demonstrates how cancer has a disproportionately high impact in nations with low SDI. For instance, the Global Cancer Observatory (GLOBOCAN) projected that by 2025, there would be about 998,000 cancer-related deaths and 1.56 million new cancer cases in India [6]. Of these, breast cancer was the most prevalent, accounting for 10.6% of all cancer-related deaths nationwide and 13.5% of new cases. Although treatment for breast cancer has advanced, many Indian women receive their diagnosis at an advanced stage because they do not use the available healthcare facilities, which results in lower survival rates (93.3% for stage I and 24.5% for stage IV). Poorer results and later diagnoses are associated with lower educational attainment. Cancer burden studies are supported by population-based cancer registries (PBCRs), which indicate that 21.8% of female cancer-related DALYs are caused by breast cancer. Only 1.6% of women in the 30–69 age range have been screened, despite the Indian government having put in place a screening program for them [7].

**Figure 1:**
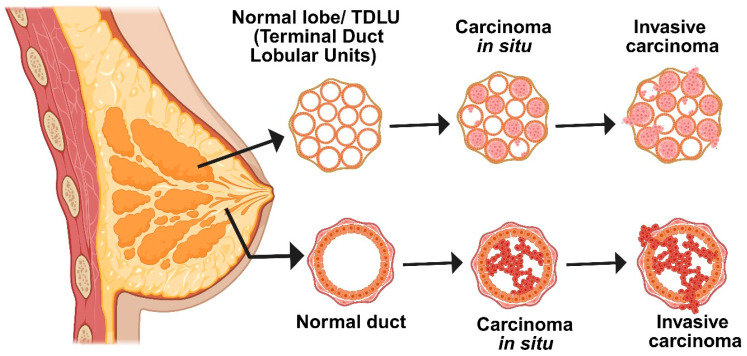
Schematic diagram representing different stages of breast cancer. Breast tissue transformation progresses from normal to carcinoma in situ, where abnormal cells proliferate but remain within the duct. The final stage is invasive carcinoma, in which malignant cells have fully penetrated the basement membrane and spread into nearby tissues.

The development of breast cancer is a slow, methodical process that is influenced by both environmental and genetic factors [8]. Normal breast cells first undergo changes such as hyperplasia or increased cell growth, which is followed by premalignant changes and, ultimately, in situ carcinoma, in which abnormal cells stay contained within the breast ducts (Figure 1) [9]. The “two-hit” model is a crucial mechanism underlying this progression: a germline genetic mutation is followed by a second mutation brought on by exposure to the environment or elevated estrogen levels, which accumulates additional genetic damage. Clusters of aberrant cells start to grow as a result of these mutations, initially manifesting as ductal hyperplasia with no obvious abnormalities [10]. These cells proliferate more quickly during the promotion phase, frequently with the help of neighboring inflammatory and stromal cells or their own growth signals. Cells develop into in situ carcinoma over time as DNA damage persists and the body’s repair mechanisms are overtaxed. Some cells eventually acquire the capacity to infiltrate neighboring tissues as a result of additional mutations and interactions within the tumor’s environment. This signifies the transition to invasive breast cancer, which is more likely to spread to other body parts. Early detection and intervention are essential due to the gradual and intricate nature of this transformation. In summary, accumulating genetic alterations and extrinsic risk factors propel the multi-step process of breast carcinogenesis [11].

Early tumor detection and reducing the negative effects of chemotherapy on healthy cells are two main problems with traditional cancer treatments. Eliminating cancerous cells while minimizing damage to healthy tissues and the immune system is the primary challenge in today’s cancer treatments. Traditional cancer treatments face challenges of early detection and minimizing chemotherapy’s harmful effects on healthy cells. Nanotechnology offers promising solutions by improving diagnosis, prevention, and therapy through nanoparticle-based systems. These nanoparticles enhance tumor targeting accuracy through two strategies: passive targeting, which exploits leaky tumor vasculature, and active targeting, which utilizes receptor-mediated binding for selective cancer cell uptake [12,13]. The active targeting strategy can target folic acid receptors, which are frequently observed to be overexpressed in breast cancer cells. The folate receptor, a glycosylphosphatidylinositol (GPI)-anchored cell surface protein, is overexpressed in most malignant tissues but exhibits restricted distribution in normal organs [14]. High levels of folate receptor expression are commonly observed in epithelial cancers of the ovary, cervix, breast, lung, kidney, colorectum, and brain, whereas sarcomas, lymphomas, and malignancies of the pancreas, testis, bladder, prostate, and liver generally exhibit low or negligible expression. Folate (pteroylglutamate) is a water-soluble B vitamin essential for DNA synthesis, methylation, and repair [15]. Its synthetic form, folic acid, is a small molecule (441 Da), stable across a wide range of temperatures and pH conditions, cost-effective, and non-immunogenic. Importantly, folic acid retains its receptor-binding affinity even after conjugation with therapeutic agents or imaging probes. Upon binding to folate receptors localized within caveolae, the complex is internalized via endocytosis. As the endosomal pH decreases to approximately 5, folate dissociates from its receptor, enabling the subsequent release of the conjugated drug [16]. The biology of folic acid, its overexpressed receptors in breast cancer, and its application in breast cancer therapeutics will all be covered in this review.

## 2. Progression of Breast Cancer

A combination of genetic and non-genetic factors influences the risk of breast cancer. Although many of these can still have genetic roots, non-genetic factors include things like age, hormone changes (like early periods or late menopause), lifestyle choices (like drinking alcohol or being obese after menopause), hormone use, radiation exposure, dense breast tissue, and some abnormal breast cell changes. Breast cancer is a collection of biologically distinct conditions rather than a single disease [17]. Tumor behavior and treatment response are shaped by variations in composition, gene activity, and environmental factors. Current clinical decisions rely on tumor size, grade, lymph node status, and biomarkers, but these often fail in advanced or resistant cancers. Advances in genome sequencing have recently enhanced the molecular classification of breast cancer, improving prospects for more precise treatment selection [18]. These technologies have provided insight into the growth, complexity, and dissemination of tumors. Researchers are also coming to the conclusion that the entire tumor environment is important and that not just the cancer cells themselves are important.

A thorough system that considers the tumor’s appearance under a microscope, clinical characteristics, and comprehensive molecular data is used to classify breast cancer. Depending on whether the cancer cells have spread outside of the ducts or lobules into neighboring breast tissue, tumors are initially classified as either invasive or in situ (non-invasive) at diagnosis (Figure 1). Ductal carcinoma in situ (DCIS) is the most prevalent in situ form; however, only 10–30% of DCIS cases develop into invasive disease, and at this time, there are no trustworthy markers to identify which ones will [19]. The group of invasive breast cancers is diverse. About 60–75% of cases are of the most common type, invasive ductal carcinoma (IDC). The second most common is invasive lobular carcinoma (ILC), which accounts for 10–15% of cases. IDC and ILC both develop in the breast, but they behave differently in terms of appearance, spread, and response to therapy. The most prevalent of IDC’s numerous subtypes is the “no special type” (NST) variation [18]. In contrast, ILC exhibits a distinct pattern in which its cancer cells lose their capacity to adhere to one another and instead move in single lines through the surrounding tissue. Less than 7% of cases of breast cancer are of less common subtypes. The Bloom–Richardson grading system assesses tumor aggressiveness based on cellular abnormality, disorganization, and division rate. Inflammatory breast cancer, though rare, is highly aggressive, spreading through the skin lymphatics and linked to greater metastatic risk and poorer prognosis.

Doctors categorize breast tumors according to their appearance under a microscope, as well as the presence of three important proteins: HER2 (a growth-promoting protein), progesterone receptor (PR), and estrogen receptor (ER). These fall into three main categories: triple-negative breast cancer (TNBC), which does not have any of the three markers, ER-positive (ER+), and HER2-positive (HER2+) [20]. Further, the ER-positive subclassified to Luminal-A and Luminal-B based on HER2 expression and Ki67 proliferation. Approximately 70% of all breast cancers are ER+ cancers. Although 10% or more is frequently regarded as more significant for treatment, a tumor is deemed ER+ if at least 1% of its cells test positive. About 15% of cases are HER2+ cancers, which can be either ER+ or ER−. Immunohistochemistry is typically used to determine HER2 status, and genetic tests such as CISH or FISH are occasionally used to check for HER2 gene amplification [21]. “HER2-low” cancers, which do not meet the requirements for complete HER2 positivity but nevertheless exhibit low to moderate HER2 expression, have attracted increasing attention recently. Newer targeted therapies might work for these. About 15% of breast cancers are TNBC, which is devoid of ER, PR, and HER2 [22]. These tumors frequently affect younger patients, have a tendency to act more aggressively, and are associated with a higher risk of recurrence and brain or lung metastases. A significant portion of breast cancer deaths is also attributable to TNBCs. These markers are still crucial for outcome prediction, treatment selection, and clinical trial design, even though they do not adequately represent the biological complexity of breast cancer.

### 2.1. Pathophysiology of Breast Cancer

**Figure 2:**
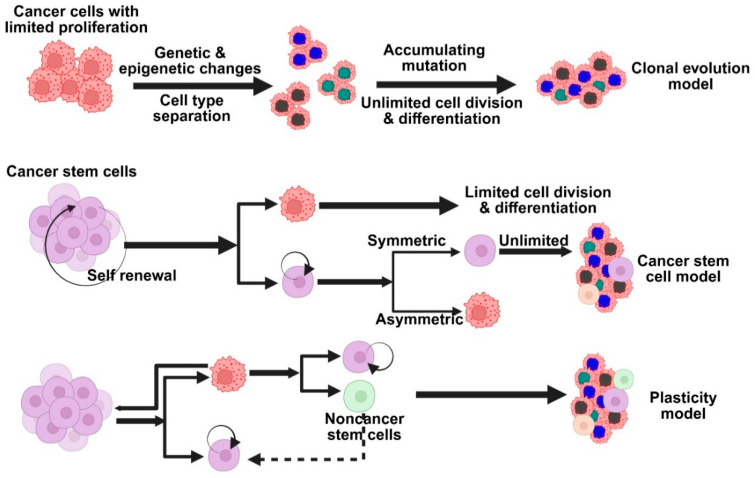
Models based on the primary hypotheses on the development of breast cancer. Different models explain tumor heterogeneity—clonal evolution, cancer stem cell, and cellular plasticity models.

The sporadic clonal evolution model and the cancer stem cell (cSC) model are the two primary hypotheses regarding the development of breast cancer (Figure 2). According to the clonal evolution model, any healthy breast cell may gradually acquire random mutations, and the cells that have the advantage of survival keep growing and proliferating until they eventually develop into tumors [23]. On the other hand, the cSC model suggests that initiating and maintaining tumor growth is the responsibility of a limited set of specialized cells known as stem cells or progenitor cells. There is no mutual exclusion between these models. Indeed, some scientists think that cancer stem cells themselves may change over time, combining the two concepts into a more adaptable tumor development model. Rare, long-lived cells in the breast called normal breast stem cells (nBSCs) have the ability to self-renew and differentiate into various breast cell types, such as luminal and myoepithelial cells, which aids in the reconstruction of the breast’s intricate structure. Specific surface markers, such as low levels of CD24, high levels of CD44, and ALDH1 expression, can be used to identify them. Interestingly, they do not express the estrogen and progesterone receptors or markers that are normally present in endothelial or blood cells [24]. These stem cells are mostly located in the breast’s ductal regions, where they are nestled just above the basal layer and encircled by progenitor cells that divide quickly. They only make up around one out of every 2000 breast epithelial cells, which makes them extremely uncommon. Research has shown that progesterone indirectly contributes to this process. It accomplishes this through the release of a signaling molecule known as RANK ligand by progesterone receptor-positive cells in the vicinity. This discovery may help us better understand how hormones affect healthy breast development and how hormone-driven signals may play a role in the development of breast cancer.

nBSCs are believed to be excellent candidates for accumulating genetic and epigenetic alterations over time because of their extended lifespan in the tissue. They may become cSCs as a result of these alterations, which may interfere with their typical self-renewal behavior. After undergoing transformation, cSCs can divide to produce more specialized progenitor cells, which in turn promote the development of different subtypes of breast cancer while simultaneously preserving the cancer stem cell pool. Another possibility is that progenitor cells, rather than stem cells, may be the source of breast cancer. Lim and colleagues provided evidence in support of this theory by demonstrating that specific luminal progenitor cells in the breasts of individuals with BRCA1 mutations may function as cancer-initiating cells, particularly in tumors that resemble the basal subtype of breast cancer [25]. This is supported by recent studies in BRCA1-deficient mouse models, which indicate that luminal progenitors rather than actual stem cells are most likely the source of both BRCA1-related and basal-like breast cancers. As a result, a third emerging theory has surfaced, according to which progenitor and stem cells are extremely flexible, or “plastic,” throughout the development of cancer [26]. This adaptability might affect the kind of breast cancer that arises, indicating that the features of the tumor might be influenced by the cells’ initial type as well as how they alter and adapt as the tumor grows. Cancer cells can change their behavior and traits in response to their surroundings or sporadic gene activity; they are not fixed into a single identity [27]. Tumor diversity is greatly influenced by this adaptability, also referred to as cell plasticity. Plasticity, which helps cells adapt and survive in changing environments, is widely regarded as a crucial component of both normal biology and cancer, despite the fact that its precise definition can vary.

Large-scale modifications to chromosome segments, known as copy number alterations (CNAs), significantly influence the genetic makeup of human cells, particularly in breast cancer [28]. CNAs involve larger changes, ranging from minor regional alterations to whole-chromosome duplications or losses, in contrast to tiny DNA mutations that only impact a few nucleotides. These changes in breast cancer are not sporadic; rather, they are a component of a multifaceted process that propels tumor growth, progression, and treatment resistance. Although current treatments are usually based on the cancer subtype of the patient, this approach is frequently less effective due to the wide variability within and between tumors. This genetic diversity is greatly influenced by CNAs, which are prevalent in invasive breast cancer [29]. CNAs are emerging as potential biomarkers for personalized therapies; however, their clinical use requires advanced detection methods and a deeper understanding of their role in tumor behavior and treatment response. The dynamic evolution of CNAs, even without driver mutations, enables breast tumors to adapt, promoting progression and drug resistance, especially under treatment pressure. For instance, resistance frequently develops as a result of the emergence of novel CNAs in ER-positive breast cancers treated with CDK4/6 inhibitors, which are currently a standard therapy [30]. This emphasizes how important it is to comprehend how, when, and why CNAs change. In order to address this, scientists have put forth a number of models that explain how CNAs arise in breast cancer.

**Figure 3:**
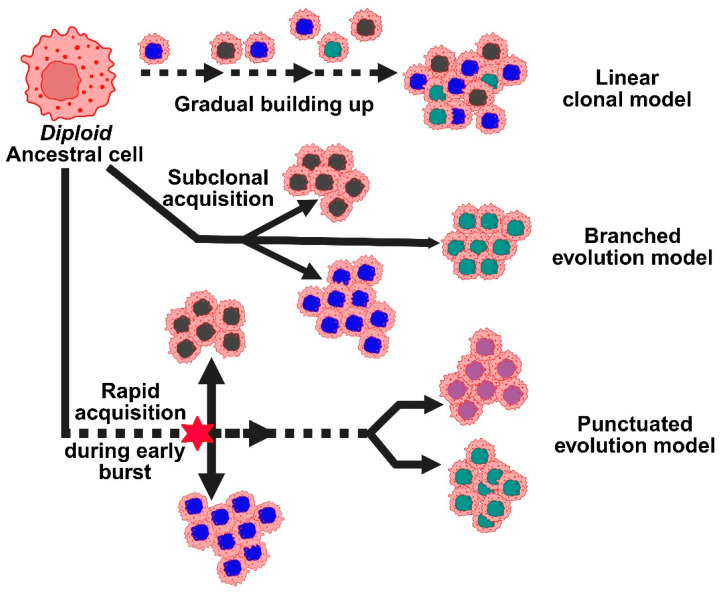
Represents the different models for copy number alterations in the progression of breast cancer. Copy number evolution models: linear, branched, and punctuated, describe how CNAs develop over time, driving tumor heterogeneity.

According to the linear clonal model, CNAs start off as a common ancestral cell and gradually build up. The tumor gains a growth advantage with each new CNA, and as the cancer spreads, the initial alterations continue (Figure 3). However, researchers developed a second model, the branched evolution model, because breast tumors frequently exhibit significant genetic variation within and between the primary and metastatic sites [31]. According to this model, distinct subclones or groups of cancer cells, each with a distinct set of CNAs, evolve independently. Another perspective is provided by a more recent concept known as the punctuated evolution model. According to this model, there is an early CNA burst during tumor development, followed by a protracted period of comparatively stable genetic material. These truncal alterations, which are early CNAs, can act as reliable indicators of the cancer’s genesis. CNAs have promise for early detection because they can show up very early, even before symptoms show up or tumors become invasive [28]. Certain CNAs may be used to direct more frequent screenings, preventive treatments, or even risk-reducing surgeries, particularly in high-risk individuals, if they are consistently associated with the early stages of breast cancer or with increased risk. Incorporating these CNA markers into population-wide screening may eventually lead to better results and earlier detection of breast cancer.

### 2.2. Tumor Microenvironment

**Figure 4:**
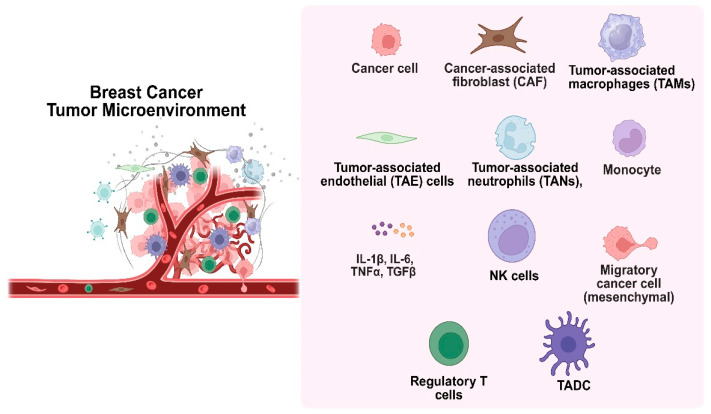
The tumor microenvironment, consisting of cellular and non-cellular elements, plays a key role in cancer development.

The development of cancer is significantly influenced by the tumor microenvironment (TME), which is made up of both cellular and non-cellular components such as stromal cells, extracellular matrix, and interstitial tissues (Figure 4). Cancer-associated fibroblasts (CAFs), mesenchymal cells, tumor-associated macrophages (TAMs), endothelial cells, and immune cells like lymphocytes and NK cells are important cellular participants [32]. Poor cancer outcomes are frequently associated with persistent, unresolved inflammation within the TME. Tumor behavior is shaped by ongoing interactions between immune cells, cancer cells, and stromal constituents. Because of their short lifespan, neutrophils were once thought to be passive participants. However, new research indicates that tumor-associated neutrophils (TANs), which are maintained by chemotactic signals from polymorphonuclear leukocytes, can actively affect the growth and progression of tumors. The most prevalent immune cells in the human bloodstream, neutrophils, are first responders to inflammation and control it by releasing chemokines and cytokines. Recent studies have shown that neutrophils, despite being thought of as a component of the body’s defense against tumors, can actually promote tumor growth and metastasis. Signals associated with tumors, such as cell lysis and genomic instability, drive their recruitment to the TME. Cytokine signaling, especially IL-8 through the NF-kB pathway, activates neutrophils in tumors with mutated K-ras [33]. By encouraging neutrophils to release arginase-1, which depletes extracellular arginine- a nutrient essential for T cell activation and proliferation, tumor-derived IL-8 inhibits T cell function. In the end, this weakens the immune response against the tumor.

G-CSF, IL-6, VEGF-α, and IL-1β are among the cytokines released by tumor and stromal cells that can cause neutrophilia and encourage immunosuppressive characteristics in TANs. Because anti-apoptotic NF-kB family proteins are upregulated in TANs, they may live longer than normal neutrophils. Tumor secretion of GM-CSF, which increases neutrophil production and triggers Oncostatin M (OSM), a cytokine that increases VEGFα expression via the Jak/STAT pathway, is a crucial interaction between TANs and tumor cells that promotes angiogenesis. By encouraging cell adhesion and inducing the production of VEGF and IL-8, hepatocyte growth factor (HGF) also aids in the remodeling of the tumor microenvironment. The PI3K and MAPK signaling pathways mediate the effects of HGF, which are essential for promoting angiogenesis, metastasis, and cancer invasion.

## 3. Conventional Therapy for Breast Cancer

Although the majority of patients with breast cancer undergo surgery to remove the primary tumor, some cancer cells frequently survive. If these remaining cells are not completely eradicated by subsequent treatments like radiation or targeted therapies, the cancer may return. Treatments that target growth receptors or hormones initially aid in slowing the cancer’s progression, but eventually, the disease may develop resistance and spread [34]. Chemotherapy becomes the primary treatment option when the disease has progressed to the metastatic stage, which indicates that it has spread outside of the breast (Table 1). Chemotherapy has serious side effects that harm healthy cells and frequently force patients to discontinue treatment early. Safe dosages are limited by the accumulation and long-term toxicity of medications such as doxorubicin and daunorubicin [35]. Although each class carries unique risks, combination chemotherapy for breast cancer typically combines an anthracycline (doxorubicin, epirubicin) or a taxane (paclitaxel, docetaxel) with other medications. Particularly in patients with advanced cancer who receive repeated cycles, anthracyclines have the ability to bind fat molecules in heart tissue, causing cumulative, irreversible cardiac damage that restricts lifetime use. Taxanes are preferred in late-stage therapy because they primarily inhibit the production of bone marrow and blood cells, but this effect is usually reversible after treatment breaks. In general, combination chemotherapy aims to strike a balance between minimizing damage to healthy organs and killing as many cancer cells as possible.

When treating metastatic breast cancer, especially when previous treatments aimed at hormones or growth signals stop working, gemcitabine and paclitaxel are sometimes combined as a chemotherapy regimen (called GT). This mix makes a safer substitute since it helps prevent the heart damage observed with anthracyclines. However, they must be administered separately in different IV formulations, as paclitaxel is not water-soluble, whereas gemcitabine is. These variations in how the drugs behave in the body, how they are absorbed, distributed, and cleared, mean they do not always reach or remain in the cancer cells simultaneously [36]. Ideally, both medications should cooperate inside the tumor to increase their total impact and lower the total dosage required, thus minimizing damage to healthy tissues. Current injectable forms make this difficult, though. Even trying to infuse them concurrently presents difficulties, including drug incompatibility and skin reactions at the injection site. Maintaining good levels of both drugs in tumors without generating notable side effects elsewhere is, therefore, quite challenging.

**Table 1:** Approved conventional chemotherapeutics that are regularly used for the treatment of breast cancer.

Drugs	Mode of Action	Limitations	Ref.
Cyclophosphamide (prodrug)	Active components Acrolein and Phosphoramide mustard (DNA alkylating agent) damage DNA by crosslinking.	Stimulate the immune system	[37]
Methotrexate (antimetabolite)	Stops tetrahydrofolate (THF) synthesis, inhibiting dihydrofolate reductase. Therefore, it stops DNA replication and protein synthesis.	Poor aqueous solubility & hepatotoxicity	[38]
Thiotepa (antineoplastic)	Alkylating (Guanine) agent involved in depurination and crosslinking DNA	Skin toxicity & myelosuppression	[39]
5-fluorouracil (5-FU) (antimetabolite/analog of uracil)	Interfere with thymidylate synthase & misincorporate metabolites in DNA & RNA.	Myelosuppression & mucositis	[40]
Capecitabine (prodrug/neoadjuvant)	Converted to 5-FU at the tumor site.	Gastrointestinal adverse event	[41]
Vinorelbine (vinca-alkaloid)	Inhibits tubulin polymerization & binds to mitotic microtubules, blocks mitosis at the G2-M phase	Granulocytopenia, thrombocytopenia, neurotoxicity	[42]
Doxorubicin (anthracycline)	Intercalates to DNA, inhibits topoisomerase II, generates ROS, damages DNA	Lower blood cell count, cardiotoxicity, healthy tissue ulceration	[43]
Docetaxel (antineoplastic)	Disorganize microtubular network, antimitotic	Febrile neutropenia, enterocolitis, bronchospasm	[44]
Paclitaxel (taxane)	Microtubule depolymerization, mitotic arrest	Myelosuppression, peripheral neuropathy	[45]
Lapatinib	Target the tyrosine-kinase domain of human epidermal growth factor receptor-2 (HER2), subdue phosphorylation, MAPK signaling, and Akt/mTOR pathways.	Skin lesions, hepatic toxicity	[46]
Trastuzumab (monoclonal antibody or mAb)	Binds with high affinity to HER2, induces cytostatic effects related to G1 arrest, reduces cell proliferation, and controls the effects of various pro-angiogenic and anti-angiogenic factors	Cardiotoxicity	[47]
Margetuximab (2nd Gen, Fc-engineered mAb)	Binds to Fab epitopes of HER2, shows Fc-independent antiproliferation, and increased binding affinity to CD16A.	Hematologic toxicity	[48]
Atezolizumab (FcγR-optimized mAb)	Interferes with binding of PD-L1 to PD-1 receptor, immunosuppressive signal reduction, and enhances T-cell-driven immunity.	Cardiovascular toxicity	[49]
Abemaciclib (LY2835219)	Selectively inhibit CDK4/cyclin D1 complex with IC50 2 nM/L & CDK6/cyclin D1 with IC50 10 nM/L	Neutropenia, fatigue, and diarrhea.	[50]
Alpelisib (BYL719)	PI3K inhibitor, specifically PI3Kα (catalytic p110α subunit)	Hyperglycemia, diarrhea, blurred vision, bladder pain, and frequent urination	[51]
Palbociclib	Inhibit CDK4 & CDK6 along with serine-threonine kinase.	Neutropenia, anemia, fatigue	[52]
Tucatinib	Involves HER2 targeting and inhibiting tyrosine kinase	Fatigue, diarrhea, and an increase in the level of ALT & AST	[53]
Pertuzumab (Humanized mAb)	Binds to the dimerized domain of HER2 and inhibits heterodimerization	Neutropenia, diarrhea	[54]
Tamoxifen (nonsteroidal antiestrogen)	Selective ER modulator, useful in ER-positive breast cancer	Vaginal dryness, Hot flashes	[55]

### 3.1. Nanomedicine in Breast Cancer

Improving patient outcomes requires treating breast cancer, particularly when it has progressed. Nanomedicine, which uses small, engineered materials to improve cancer therapy, has become a promising strategy in recent years. Drug resistance, adverse effects, and the spread of cancer to other body parts are some of the main challenges faced by traditional treatments like radiation, chemotherapy, and surgery [56]. These techniques may also have issues with ineffective drug delivery, inaccurate targeting, and detrimental effects on healthy tissue. In order to overcome these obstacles, researchers have created a number of FDA-approved nanomaterials, such as metal-, polymer-, and smart nanoparticles, which enhance the body’s ability to absorb, target, and distribute medications [57]. These developments are meant to help patients receive less harmful and more effective treatments. Biocompatible, easily modifiable, and efficient drug delivery are just a few advantages of biogenic nanoparticles like liposomes. Designing liposome-based nanoparticles that more effectively target tumor sites is made possible by their easily modifiable surfaces. In the meantime, cubosomes and polymeric nanoparticles are notable for their capacity to transport a variety of substances, whether they are amphiphilic, non-polar, or water-soluble, and release them at the tumor site in a pH-sensitive, controlled manner while remaining biodegradable [58].

Likewise, exosomes possess the exceptional capacity to penetrate cell membranes and transport therapeutic molecules, such as proteins or nucleic acids, precisely where they are required for the treatment of breast cancer [59]. Additionally, dendrimers are highly versatile in nanomedicine because of their high water solubility, biodegradability, and ease of linking to targeting agents. Among the most stable kinds of metallic or LSPR-based nanoparticles are gold nanoparticles [60]. They are excellent for attaching targeting molecules because they are simple to alter on the surface. Because of their size and biocompatibility, these particles provide strong binding, accurate targeting, quick delivery, a lengthy half-life in the body, and improved tumor uptake. Due to their dual use in therapy and imaging, magnetic nanoparticles also exhibit a lot of promise. They can more readily penetrate the membranes of breast cancer cells when coated with polyarabic acid [61]. Additionally, they give researchers more control over drug delivery by enabling the reversible loading of chemotherapy drugs. In the context of nanomedicine or drug delivery systems, passive targeting and active targeting of breast cancer cells for the delivery of chemotherapeutics are gaining potential at the present time with the help of nanoengineering.

### 3.2. Potential Nanocarriers in Breast Cancer Therapy

**Figure 5:**
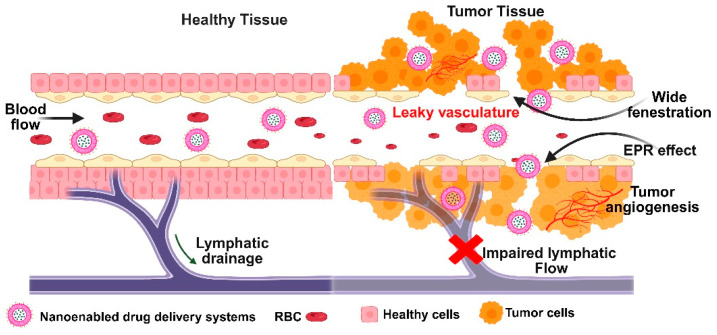
Schematic depiction of enhanced permeability and retention effect in tumor tissue, highlighting leaky vasculature and tumor angiogenesis.

Emerging as essential instruments in the multimodal therapy of cancer, nanoparticles greatly increase the efficacy of chemotherapy, immunotherapy, radiotherapy, and gene therapy [62]. By extending systemic circulation time and minimizing off-target toxicity by preferred accumulation in tumor tissues via the enhanced permeability and retention (EPR) effect (Figure 5), nanoparticulate drug delivery systems improve pharmacokinetic and pharmacodynamic profiles in chemotherapy [63]. Employing targeted drug delivery made possible by these nanocarriers raises therapeutic efficacy and minimizes systemic side effects. Within the field of immunotherapy, which is rapidly developing in oncology, nanoparticles are under investigation for their ability to directly deliver immunomodulating agents to the tumor microenvironment, thus enhancing immune responses and coordinating with other treatment modalities. Allowing the focused delivery of radionuclides or radiosensitizers, increasing tumor-specific radiation doses, and sparing healthy tissue contribute to enhanced therapeutic accuracy and safety in radiotherapy [64]. This method, especially in combination with image-guided radiotherapy methods, enables exact localization of radiation distribution, lowering collateral damage to nearby normal cells and enhancing clinical results. By allowing nanoparticle-based delivery systems, nanotechnology is greatly helping to advance gene therapy for cancer. Easy manufacturing, functionalizing, low immunogenicity, and low toxicity are just a few of the benefits these nanoparticles present. High production costs, scalability, safety, and the complexity of nanosystems restrict their clinical implementation despite their promise in several respects [64]. The physicochemical characteristics of nanoparticles define their biocompatibility and toxicity; hence, strict manufacturing and characterization techniques are required. Safety issues also surround possible long-term health and environmental hazards, including the production of reactive oxygen species that cause inflammation, toxicity, and perhaps damage to neural or dermal tissues. Consequently, nanotoxicology has become a major focus of study to assess and control these hazards in applications of cancer treatment.

To make treatment more effective and reduce side effects in breast cancer therapy, Li et al. developed a cationic liposome system that carries three agents together: paclitaxel, crizotinib, and Bcl-xL siRNA [65]. These liposomes showed strong tumor-targeting ability due to the EPR effect. Lab tests revealed that about 64% of crizotinib and 55% of paclitaxel were released within 12 h, and they were released simultaneously. When tested on breast cancer cells (MCF-7), the combination of crizotinib and paclitaxel worked better together than alone, showing a synergistic effect. The liposomes were efficiently taken up by the cells, and the siRNA effectively reduced Bcl-xL protein levels, which are linked to cancer cell survival. Overall, this co-delivery system significantly increased the cancer-killing effects in vitro, highlighting its strong potential as a breast cancer treatment strategy [65]. Yildirim and colleagues developed smart polymeric nanoparticles loaded with naringenin to boost their bioavailability and effectiveness against breast cancer [66]. These nanoparticles were designed to respond to both temperature and pH changes using specific monomers, N-isopropyl-acrylamide for heat sensitivity and vinyl-imidazole for pH sensitivity. They examined how the drug was released, as well as its impact on breast cancer cell growth, cell death, apoptosis, and the cell cycle. The formulations were found to be stable and effective at killing breast cancer cells by the apoptotic pathway while remaining non-toxic to healthy human epithelial cells [66]. Liu et al. developed a smart drug delivery system combining pyroptosis, cancer starvation, and chemotherapy to target breast cancer [67]. It used glucose oxidase-linked iron oxide nanoparticles loaded with paclitaxel and a pro-apoptotic plasmid, all encapsulated in PLGA and coated with aptamer-modified chitosan for targeted delivery. The system showed pH-sensitive drug release and significantly reduced cancer cell viability (to 12.1%) compared to non-targeted treatments. It demonstrated strong synergistic effects in vitro and minimal side effects in vivo, highlighting its therapeutic potential [67]. Several other nanoparticulate systems (Table 2) have been researched to deliver chemotherapeutics to breast cancer cells.

### 3.3. Passive Diffusion

Using either convection or diffusion, passive targeting introduces nanocarriers into tumors via the tumor tissue’s leaky blood vessels. When there is no net fluid flow, convection is the predominant method for large molecules to pass through large pores. On the other hand, diffusion, which depends on concentration gradients and does not require cellular energy, is primarily used by small molecules like oxygen. Diffusion is the main mechanism for drug transport in tumors because convection is restricted by the high pressure in the interstitial space of the tumor [81,82]. Through the Enhanced Permeability and Retention (EPR) effect, which has emerged as a crucial tactic in cancer-targeted drug design, nanocarriers and medications selectively accumulate in tumors. Although it is less effective in poorly vascularized cancers like prostate or pancreatic cancer, this effect is applicable to the majority of rapidly growing solid tumors. Nanocarriers must avoid the immune system and stay in circulation for long periods of time in order for the EPR effect to function as best it can. This allows drug levels at the tumor site to rise dramatically, up to 10–50 times higher than in normal tissue in just 1–2 days [83]. To do this, nanocarriers should have three essential characteristics: (i) a neutral or negatively charged surface to minimize kidney elimination; (ii) a size between 10 and 100 nm to avoid kidney filtration and liver clearance while still passing through tumor blood vessel gaps; and (iii) the capacity to evade recognition and destruction by the reticuloendothelial system [84,85]. Passive targeting has a number of drawbacks despite its benefits. (i) The degree to which a tumor is vascularized and the amount of angiogenesis—the formation of new blood vessels, varies depending on the type and location of the tumor. This affects the ease with which nanocarriers can enter the tumor from the bloodstream. (ii) Drug uptake and even distribution are hampered by high interstitial fluid pressure and inadequate lymphatic drainage in solid tumors [86,87]. The EPR effect favors larger, long-circulating nanocarriers because they are more likely to be retained in the tumor, whereas smaller molecules tend to diffuse away more readily. This pressure also explains why.

### 3.4. Active Targeting

By employing molecules that bind to receptors that are highly expressed in cancer cells, active targeting helps overcome the drawbacks of passive drug delivery by facilitating drug entry through receptor-mediated endocytosis. Drug resistance mechanisms such as the P-gp efflux pump can be circumvented using this technique. These particular molecules are added to nanocarriers to improve tumor targeting [88]. In order to be effective, active targeting agents should primarily build up in tumors, be readily connected to nanocarriers that target cell surface receptors, relate to characteristics of cancer such as drug resistance, and simplify drug delivery procedures. Anticancer medications can be precisely guided to the appropriate receptors on target cell membranes by nano-drug delivery systems that contain particular functional groups, such as peptides, folic acid, and antibodies. By aiding in the binding of the nano-enabled delivery system to tissues, these groups cause endocytosis, which allows the drug to enter particular cells. The interaction between the surface groups of the delivery system and cell membrane receptors is necessary for this targeted delivery. Because breast cancer is a complex and heterogeneous disease, active targeting in treatment presents significant challenges [89]. Tumor variability, the development of resistance, restricted drug penetration, off-target effects, and immune responses to targeting agents are among the difficulties. Variations in target expression between patients and tumor subtypes exacerbate these problems. The TME of breast cancer is made up of various interacting cells that affect how well a treatment works, such as immune cells and cancer-associated fibroblasts. Different TME profiles are displayed by subtypes such as Luminal B, HER2-positive, and TNBC.

TME can be categorized as either immunosuppressive or immunoreactive based on its impact on the immune response. Tumor-infiltrating lymphocytes (TIL), which are the main immune cells within the TME, are diverse and found in both tumor nests and the surrounding stroma. In solid tumors, TILs primarily consist of CD3^+^ T cells and, to a lesser extent, CD20^+^ B cells. The CD3^+^ T cells include CD8^+^ cytotoxic T cells, CD4^+^ helper T cells, and Foxp3^+^ regulatory T cells [90]. Each of these subtypes contributes differently to immune regulation and plays distinct roles in modulating the immune response in breast cancer. During the progression of TNBC, the tumor immune microenvironment or TIME undergoes significant remodeling, marked by shifts in immune cell ratios and the release of various immune-suppressive and immune-activating cytokines. Based on these microenvironmental components, TNBCs are categorized into different TIME subtypes, which help predict patient outcomes and guide the development of targeted therapeutic strategies tailored to each subtype’s unique characteristics [91]. Therapeutic efficacy may be increased by focusing on TME elements like immune checkpoints and cancer-associated fibroblasts. Modern technologies such as single-cell sequencing and spatial genomics, which aid in deciphering both intrinsic genetic factors and extrinsic TME influences, are increasingly guiding personalized treatments and immunotherapy strategies.

Many different kinds of cells have the receptor EGFR on their surface, but breast cancer cells have a disproportionately high amount of it. Research has indicated a strong correlation between the growth and metastasis of cancer cells and elevated EGFR levels [92]. In one study, Wan and associates used a novel method to create a targeted cancer treatment: they applied a platelet membrane to dendritic mesoporous silica nanoparticles that were loaded with lapatinib and chlorin e6 [93]. This platform was created to treat breast cancer by combining EGFR inhibition with photodynamic therapy (PDT). The fundamental concept of PDT involves a nonthermal photochemical reaction that occurs only when three components, a photosensitizer, oxygen, and visible light, are present simultaneously. Tests on animals and in the lab demonstrated that this system could successfully deliver medications straight to breast tumors. Furthermore, the treatment assisted in directing additional nanoparticles to the tumor site after PDT damaged the blood vessels in the tumor, which improved drug accumulation. By suppressing EGFR activity, this ongoing targeting eventually slowed the growth of the tumor and stopped it from spreading [93].

Tumor vascularization provides the blood supply necessary for the tumors’ quick growth and metastasis during the progression of the tumor. Targeting tumor angiogenesis is one of the active targeting strategies that has seen a surge in research in recent years. Many tumor cells overexpress the angiogenic protein VEGF, which increases the vascularization and vascular permeability of tumor tissues. As a result, VEGF targeting has garnered a lot of interest [94,95].

Sen et al. show that by interfering with the Notch and RTK or receptor tyrosine kinase pathways, Axitinib and LY411575 (a γ-secretase inhibitor) efficiently target TNBC [96]. Significant apoptosis, G2/M cell cycle arrest, decreased cancer stemness, and spheroid formation are all brought on by the co-treatment. Additionally, it lowers mesenchymal and pro-oncogenic protein levels (e.g., pEGFR, pMAPK, pFAK, NF-κB) and raises the expression of the epithelial marker E-cadherin. Moreover, it inhibits the expression of pericyte markers, indicating that the epithelial-to-pericyte transition (EPT) is inhibited. Overall, the study shows how co-therapy may affect TNBC’s EMT (epithelial to mesenchymal transition) and EPT [96]. The ErbB family of receptor tyrosine kinases includes the human epidermal growth factor receptor 2 (HER2). It is an efficient target for clinical treatment of breast cancer and is highly expressed in approximately 30% of breast cancers and 20% of ovarian cancers [97]. A pH-sensitive targeted niosomal system was created by Saharkhiz et al. to deliver the anticancer medication palbociclib only to cells that overexpress HER2 [98]. They accomplished this by precisely targeting Trastuzumab molecules with quantum dots. Their drug release followed a two-stage, pH-responsive pattern, and they were able to achieve a high drug encapsulation rate of about 86%. These targeted nanoparticles dramatically increased cancer cell death, particularly in HER2-positive cells, according to cell viability tests. The successful absorption of the nanoparticles by the target cells was further validated by fluorescence imaging. All things considered, the formulations exhibit great promise as a customized approach to treating HER2-positive breast cancer [98].

A membrane-bound protein called fibroblast growth factor receptor 3 (FGFR3) is overexpressed in 20% of cases of advanced bladder cancer and aids in the development of aggressive tumors. A number of FGFR inhibitors are currently approved by the FDA to treat cancer. By developing a biodegradable pseudoconjugate polymer (PSP) with sonodynamic properties, Yang et al. recently presented a novel way to increase the effectiveness of Erdafitinib in the treatment of bladder cancer [99]. When activated by ultrasound, these nanoparticles that contain Erdafitinib produce reactive oxygen species that promote antitumor immune responses and cause immunogenic cell death. In animal studies, the formulation shows stronger tumor suppression and more effective downregulation of FGFR3 expression than Erdafitinib alone. Additionally, it complements aPD-L1 immunotherapy, providing a promising therapeutic option, particularly for patients with immune-cold bladder tumors [99]. Chemotherapy failure is largely caused by tumor cells’ multidrug resistance (MDR). P-glycoprotein (P-gp), an energy-dependent transporter that actively pumps a variety of structurally diverse compounds out of cells, is a known mechanism behind MDR [100]. P-gp is frequently overexpressed in drug-resistant tumor cells, which lowers the intracellular concentration of therapeutic agents. In response, P-gp antibody-modified nano-drug delivery systems have been created, providing a focused method for efficiently detecting and treating resistant cancer cells.

## 4. Folic Acid-Driven Targeting

Numerous plant and animal-based foods contain folate, the naturally occurring and water-soluble form of vitamin B9. Because of its higher bioavailability, folic acid, the synthetic form of folate, is used in supplements and food fortification. Folate plays a critical role in the maturation and proliferation of both healthy and malignant cells because it is necessary for nucleotide biosynthesis and DNA repair through uracil methylation. Although excessive consumption may promote atherosclerosis through increased cell proliferation, it also helps regulate homocysteine, which may reduce the risk of cardiovascular disease and stroke. Because neural tube defects like spina bifida and anencephaly are closely linked to folate deficiency, women of childbearing age should take folic acid supplements on a regular basis. Megaloblastic anemia is another consequence of deficiency.

Folate receptors (FR) are approximately 35–40 kDa glycoproteins with four isoforms (FRα, FRβ, FRγ, and FRδ) that share about 70% of their sequence [101]. Glycosyl-phosphatidylinositol (GPI) anchors hold FRα, FRβ, and FRδ to the membrane, while FRγ does not. FRα and FRβ on the cell surface are the main mechanisms by which folic acid (FA) is absorbed. The high-affinity binding of FRα and FRβ to FA and its reduced forms (e.g., methyltetrahydrofolate; Kd ~10^−10^ M) is similar, despite slight variations in their C-terminal regions. Internalization and cytosolic release result from FA binding, which starts receptor-mediated endocytosis. According to structural analyses, FR takes on an open conformation at neutral pH (7.4), which makes it easier for FA to bind through important residues like Asp97, Trp154, His151, and Ser150 [102]. Following endocytosis, a conformational shift to a closed state is triggered by the acidic environment (pH 5.6–7.2), which facilitates the release of folate. Different structural regions allow the folate molecule to interact with its receptor: the γ-carboxyl group of the glutamate tail is partially solvent-exposed, the 4-aminobenzoate group interacts hydrophobically in the central region, and the pterin ring binds deep within the receptor’s active site. The γ-carboxyl group is the preferred site for chemical modification and conjugation because of this exposure and its chemical accessibility, which preserves the receptor-binding affinity of folate [102,103]. Converting this carboxylic acid into an N-hydroxysuccinimide ester, which easily forms stable amide bonds with primary amines, is a common conjugation technique. Although the pterin amine may be reactive in theory, its nucleophilicity is diminished by electron delocalization within the pterin ring, which makes direct conjugation less feasible. Furthermore, altering the pterin amine considerably reduces folate’s affinity for its receptor, which makes it an unfavorable location for drug attachment.

Folate supplementation around conception is essential to preventing birth defects because FRα plays a crucial role in embryonic development, especially in neural tube formation. Its high expression in the yolk sac indicates that it plays a role in the early neurulation process of maternal folate delivery. Its vital role is demonstrated by the fact that in mice, deletion of the Folr1 gene (which is comparable to human FOLR1) results in embryonic lethality [104]. However, dietary folinic acid supplementation can partially reverse this lethality, allowing embryos to survive with possible cardiovascular and neurological abnormalities. Due to its limited expression and lower folate transport efficiency when compared to other transporters, such as the reduced folate carrier, FRα appears to play a smaller role in normal human tissues after embryogenesis. By encouraging cellular migration, invasion, and proliferation, FRα is linked to the advancement of cancer. In a number of preclinical models, its overexpression is associated with tumor aggressiveness. It has been demonstrated that ovarian cancer cells treated with anti-FRα single-chain intrabodies exhibit decreased cellular adhesion, decreased anchorage-independent growth, and suppressed cell division [105].

Similar to this, FRα knockdown via short hairpin RNA hinders invasion, migration, and proliferation, most likely due to decreased expression of E-cadherin. Cell viability is also decreased in TNBC when FRα is silenced with siRNA. Additionally, by activating the JAK-STAT signaling pathway, folic acid can promote cell proliferation in FRα-overexpressing cells. A possible therapeutic target, FRα, is extensively expressed on the surface of tumor cells in cancers like breast, ovarian, TNBC, endometrial, mesothelioma, and lung cancers [106]. Targeted nanoenabled drug delivery is becoming a powerful tool in breast cancer treatment, helping to make therapies more effective while reducing harmful side effects. One promising approach uses specific receptors on cancer cells, like the folate receptor, as a kind of address tag to deliver drugs directly where they are needed. Because breast cancer cells have more folate receptors than healthy ones, researchers have designed special drug carriers immobilizing FA on carriers to promote small drug delivery vehicles- FA combinations that home in on these receptors.

### 4.1. Methods of FA Linking on Nanocarriers

Metal or metal-oxide nanoparticles can be made either positively or negatively charged by attaching special molecules called spacers or ligands that contain amine or acid groups. These ligands not only help control the nanoparticle’s charge but also make it water-soluble, which is an essential feature of a drug delivery vehicle. In building larger molecular structures, scientists often explore noncovalent interactions like hydrogen bonds or electrostatic forces, which are weaker but more reversible than covalent bonds. While covalent bonds are stronger, they can be problematic in biological systems. For example, when proteins attach covalently to nanoparticles, they often lose their shape and function [107]. Similarly, if a drug molecule is bound too tightly through a covalent bond, especially at its active site, it might not work unless that bond is broken first. Fortunately, the body has enzymes in the blood that can sometimes break these bonds. Still, for targeted drug delivery, using noncovalent interactions to attach drugs or targeting moieties to nanoparticles is often a smarter choice, as long as the bond is strong enough to keep the drug or targeting molecule in place until it reaches its target and binds. To easily attach surface groups like targeting agents or drugs or as whole payloads to nanocarriers, it is important to use linkers that have adaptable functional groups like thioether (R-S-R) including sulfide, sulfoxide (R-S(=O)R), or thioketal (R_2_C(SR)_2_), carbamate (RN(C=O)OR, amine, hydrazine, hydroxyl (–OH), disulfide, acetyl-hydrazone, and carbodiimide. One widely used technique for this is the EDC-NHS crosslinking method. This approach helps form stable amide bonds by linking carboxylic acid groups (–COOH) to primary amines (–NH_2_), making the conjugation process more efficient and reliable.

Farani et al. worked with iron oxide nanoparticles about 6 nm in size and encapsulated them with polyglycerol-containing hyperbranching, then attached FA to it by generating a covalent bond utilizing hydroxyl groups of polyglycerol [108]. This combination helped keep the particles from aggregating, making them more stable. The FA-conjugated particles showed a curcumin loading capacity of 88%. When tested on HeLa cells and mouse fibroblasts, the FA-conjugated Fe_3_O_4_ was taken up more by the cells compared to those without FA. FA-conjugated Fe_3_O_4_ showed promise for delivering cancer drugs. Also, when HeLa cells were exposed to these nanoparticles, there was a noticeable drop in MRI signal, especially with the folic acid-coated ones, which could be useful for cancer diagnosis [108]. Fathima et al. synthesized folic acid-loaded chitosan nanoparticles through an amide bond, and the final fine-tuned particles were about 180 nm in size, had a strong positive charge (+52 mV), and were able to hold 90% of the folic acid [109]. Importantly, when they treated Caco-2 cells with these nanoparticles, the cells stayed healthy and even took in more folic acid after 2 h compared to just plain folic acid. In tests on rats, giving them these nanoparticles orally boosted the amount of folic acid, and this suggests that chitosan nanoparticles could be a great way to deliver folic acid as a supplement combating disease conditions [109].

Rostami et al. focus on making biodegradable nanoparticles to deliver letrozole more effectively [110]. The PCL-co-PEG nanoparticles were loaded with letrozole, and then attached FA to these particles by NHS-mediated coupling to help target breast cancer cells that have folate receptors. Studies showed that the FA-coated particles were more toxic to breast cancer MCF-7 cells than the non-coated ones. They worked by turning down a protein that helps cancer cells survive (Bcl2), boosting one that promotes cell death (caspase 8), and pushing more cancer cells into a stage where they die. Overall, this biodegradable, pH-sensitive nanoparticle system with folic acid looks promising for targeting breast cancer more precisely while reducing side effects [110]. Jafarpour et al. developed a targeted drug delivery system using mesoporous silica nanoparticles (MSNs) that were modified with a copolymer made from poly(acrylic acid-co-allylamine) or PAA, which was further linked to folic acid [111]. They started by creating MSN-SH through a post-grafting modification process. Then, they carried out radical polymerization using MSN-SH as a chain-transfer agent, resulting in M-PAA, a compound rich in reactive amine and carboxyl groups. To target specific cells, folic acid was chemically attached to these amine groups through an amidation reaction, using EDC and NHS as coupling agents. When methotrexate was loaded into this delivery system, it showed strong cytotoxic effects against MCF-7 breast cancer cells. This suggests that folic acid helps the system specifically target the cancer cells by binding to their folic acid receptors [111].

Folic acid is often attached to nanoparticles using a carbodiimide-mediated reaction, commonly known as EDC/NHS amide coupling. Carbodiimides act as “zero-length” crosslinkers, meaning they help form a direct covalent bond between carboxyl groups and primary or secondary amines without becoming part of the final amide linkage. In this process, the reaction occurs between a carboxyl group and an amino group, for instance, between the amino group of folate and the free carboxyl group of a protein. Various carbodiimide reagents can be used, including N-(3-dimethylaminopropyl)-N-ethylcarbodiimide (EDC), N,N-dicyclohexylcarbodiimide (DCC), and Ethyl-3-(3-dimethylaminopropyl) carbodiimide (EDAC). As an example, Zhang et al. developed folic acid-functionalized gold nanoparticles through a three-step method [112]: first, the thiol group of glutathione binds to the gold surface via an Au-S bond; second, DCC and NHS activate the carboxyl groups of glutathione, creating a reactive intermediate; and finally, these activated groups react with the free amino group in folic acid, producing folic acid-conjugated nanoparticles.

The functionalization of drug nanocrystals with folic acid was performed by Fuster et al. in two main steps to produce FA-nanocrystals [113]. In the first step, the carboxyl groups of FA were activated, enabling covalent attachment to the nanocrystals through the –NH_2_ groups present on their surface. Specifically, 15 mg of FA, 3.75 mg of N-hydroxysuccinimide, and 2.25 mg of EDC were dissolved in 3 mL of Milli-Q water to initiate the activation reaction. This solution was magnetically stirred for 40 min. In the second step, 3 mL of chitosan-coated nanocrystals was added to the activated FA solution, and the mixture was stirred for 24 h, allowing the conjugation process to complete. Click chemistry provides a rapid, reliable, and highly efficient method for joining molecules together. One of its most famous reactions is the copper-catalyzed azide–alkyne cycloaddition (CuAAC), which links azides and terminal alkynes to form stable 1,2,3-triazoles. In a work, alkyne-modified folic acid was linked to azide-functionalized gold-coated Fe_3_O_4_ magnetic nanoparticles using the CuAAC reaction by Shen et al. [114]. Specifically, 0.01 mmol of alkyne-FA was dissolved in DMSO and mixed with the nanoparticle suspension. After a quick vortex, copper sulfate (5 mol%) and ascorbic acid (10 mol%) were added, and the mixture was stirred for 48 h. The resulting product was then collected magnetically and washed with DMSO. Click chemistry is also a popular method to conjugate FA on the surface of functionalized nanoparticles.

The loading and release of therapeutics chemistry is important for keeping therapeutic or imaging agents encapsulated in nanocarriers so that they are more soluble, stable, and bioavailable. The size and fabrications of nanocarriers, as well as intramolecular interactions like hydrogen bonding, affect how well drugs are loaded and released. Efficient loading makes sure that the drug gets to the right place, reduces side effects, and helps the drug get into the tumor better. Nonetheless, achieving low drug loading, specifically below 10%, presents a significant challenge, which can be mitigated through the utilization of nanoporous materials, drug-carrier conjugation, targeting molecules such as folic acid, or carrier-free systems. Kumar et al.’s study looks at redox-responsive polymeric nanoparticles made from a random multiblock copolymer that can deliver doxorubicin to breast cancer cells [115]. The polymer was made by ring-opening and isomerization polymerization, and folic acid was added to make it targetable. These nanoparticles, which were about 110 nm in size, were able to hold a lot of drugs (about 22%) and release them in a way that was sensitive to redox conditions. For example, they released about 72% of the drug when the pH was 5.5 and the glutathione level was 10 mM, but only 18% when the pH was 7.4. In vitro assays demonstrated increased cellular uptake (22%) and elevated apoptosis (80%) in dual-targeted systems compared to non-targeted systems. In vivo, the formulation resulted in 91% tumor regression with minimal toxicity, indicating that these redox-responsive nanocarriers are highly effective for the sustained release of drugs, as well as targeted and efficient cancer treatment [115].

### 4.2. Folate-Mediated Breast Cancer Cell Targeting

**Figure 6:**
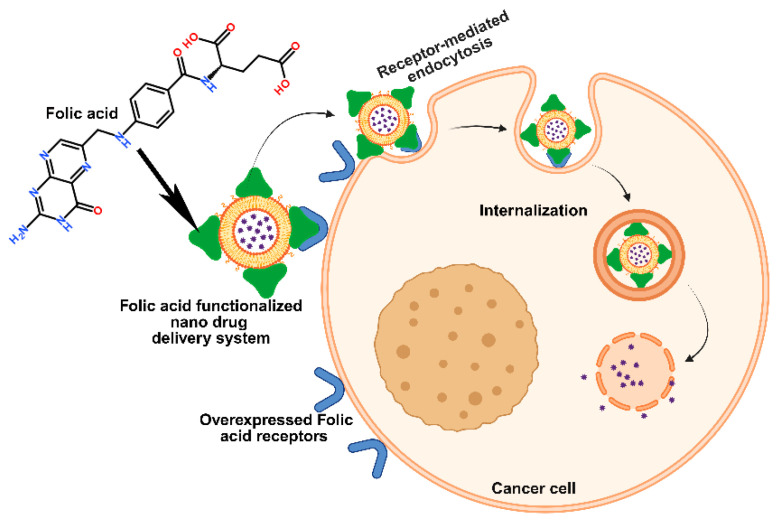
Pictorial strategy to target breast cancer cells via folic acid receptor-mediated endocytosis by folic acid-functionalized nano drug delivery systems.

The folic acid receptor has emerged as a promising target in cancer therapy because it is often overexpressed on certain cancer cells, including those in breast cancer (Figure 6). By attaching folic acid to nanocarriers, researchers can design drug delivery systems that specifically seek out and bind to these receptors. This targeted approach increases the likelihood that the nanocarriers will deliver anticancer drugs as well as imaging agents directly to tumor cells, where folic acid receptors are abundant, while largely sparing healthy cells, which have fewer of these receptors. Pandit et al. developed composite nanoplatforms composed of Fe_3_O_4_-loaded dual metal–organic frameworks (ZIF-8/-67), which were functionalized with FA and loaded with the anticancer compound quercetin [116]. The Fe_3_O_4_ component served as a contrast agent for magnetic resonance imaging, while the FA enabled selective targeting of folic acid receptor-positive breast cancer cells, specifically the MDA-MB-231 cell line. Upon delivery, the nanocomposite induced substantial reactive oxygen species production and nuclear fragmentation, ultimately triggering cancer cell death. These findings highlight the potential of the Fe_3_O_4_/ZIF-8-ZIF-67/FA/quercetin system as a promising theranostic platform, combining both imaging and therapeutic capabilities, for targeted breast cancer treatment [116]. Rodero and colleagues explored an effective way to deliver rapamycin (a drug that blocks mTOR proteins) to breast cancer cells by packaging it into nano lipid-based particles or NLCs [117]. To make these particles more precise in targeting breast cancer cells, they added FA, which helps guide the rapamycin to cells with folate receptors, which are common in many breast cancers. They created both an FA-modified version (FA-NLC-rapamycin) and an unmodified one (NLC-rapamycin), both about 100 nanometers in size with a strong negative charge and high drug-carrying capacity (about 95% and 86%, respectively) [117]. In vitro tests showed that the FA-modified version entered cancer cells more efficiently than normal cells and effectively reduced cancer cell growth, much like the standard rapamycin solution. This highlights the promise of FA as a targeting tool in breast cancer drug delivery.

Nabawi et al. developed a formulation process that involved creating PEGylating carbon nanotubes or CNTs, linking FA, and then loading the cancer drug sorafenib [118]. The MTT assay showed that the formulation was three times more effective at killing TNBC cells compared to regular sorafenib. Flow cytometry confirmed that it triggered significantly more cell death through both apoptosis as well as necrosis. It also suppressed the cancer-related protein BCL-2 more effectively while having a milder effect on cytochrome c and caspase-3, -8, and -9 [118]. In animal studies, the engineered formulation outperformed free sorafenib, boosting its bioavailability eightfold and extending its half-life by three times. Safwat developed bioinspired casein nanoparticles loaded with caffeic acid and functionalized with FA to target breast cancer [119]. When tested in rats with induced tumors, these nanoparticles led to a notable drop in key cancer and oxidative stress markers, including carcino-embryonic antigen (CEA), carbohydrate antigen 15–3 (CA 15–3), and malondialdehyde, while boosting levels of the antioxidant enzyme SOD. Varvara et al. engineered fluorescent, biocompatible carbon dots by modifying them with a biocompatible amine compound and attaching FA via a PEG linker to target breast cancer cells more precisely [120]. These carbon dots proved effective as bioimaging tools and showed strong photothermal activity when exposed to NIR light. When loaded with the Dox, the system enabled NIR-triggered drug release, increasing by 50% after localized NIR stimulation. In this study, the combined effect of drug delivery and NIR exposure significantly enhanced antitumor activity against breast cancer cells MCF-7 and MDA-MB-231, offering a promising approach for targeted and controllable breast cancer therapy [120].

Out of all the targeted strategies for breast cancer therapy that have been compared, targeting with folic acid is by far the simplest, most selective, and most translatable into the clinical arena. Antibody–drug conjugates are well-established clinically, but there are production costs, manufacturing complexity, and immune reaction issues associated. Although aptamers and peptide ligands provide excellent specificity, they remain inherently unstable, prone to enzymatic degradation, and have low to moderate tumor penetration [121]. Folic acid, a small, naturally occurring vitamin, binds efficiently and selectively to the overexpressed folate receptor-α on breast cancer cells with lesser binding to normal tissues. This allows accurate delivery of drugs with decreased off-target toxicity. Its small size not only enables it to infiltrate into the tumor better, but also allows it to undergo receptor-mediated endocytosis rapidly and facilitates the internalization of drugs. Therapeutically, folic acid conjugation is chemically simple and cost-effective and can be used in many different types of nanocarrier systems without affecting their carrier structural integrity or activity. Therefore, folic acid targeting represents the best combination of efficacy, safety, scalability, and suitability for regulatory approval for breast cancer drug delivery when compared with alternative ligand-directed approaches [122].

### 4.3. Folic Acid-Conjugated Nanostructures for Therapeutic Benefit

Numerous nano-enabled drug delivery systems are optimized today for targeted breast cancer therapy [123]. Mesoporous silica nanoparticles/chitosan/folic acid (MSNs-CS-FA) nanoformulations were assessed by Barzelighi et al. as recombinant Azurin (rAzu) carriers [124]. In contrast to other formulations, the rAzu-MSNs-CS-FA complex demonstrated pH-responsive and sustained release, with a slower rate of rAzurin release targeting FA receptors. In vitro, the formulation caused apoptosis, DNA fragmentation, and increased rAzurin uptake while inhibiting MCF7 breast cancer cells in a concentration-dependent manner. Immunologically, it suppressed IL-6, differentially regulated TLR genes, and increased the secretion of TNF-α, IFN-γ, and IL-4. In addition to downregulating tlr2/4/9, tlr3 was upregulated. Tumor volume decreased and regressed after 21 days of treatment in vivo in BALB/c mice, demonstrating successful targeted delivery [124]. Altogether, rAzu-MSNs-CS-FA showed immunostimulatory, cytotoxic, and apoptotic properties, making it a viable therapeutic option for breast cancer. In order to combat multidrug-resistant breast cancer, Okuyucu et al. created the multifunctional nanostructured lipid carrier (NLC)-based system (DOX-OA-VERA-AuNRs)@NLC, which combines chemotherapy and photothermal therapy (PTT) [125]. The PTT relies solely on light, typically in the NIR range, to achieve deep tissue penetration and destroy targeted cells or tissues through localized heat generation. To improve loading efficiency and reduce burst release, doxorubicin (DOX) was ion-paired with oleic acid (OA) in a nano-in-nano design. Gold nanorods (AuNRs) were used as photothermal agents, and verapamil (VERA) was co-loaded as a chemosensitizer to overcome drug resistance. In vitro, it was demonstrated that NIR triggered drug release, allowing for site-specific and synergistic chemo/photothermal treatment [125]. Additionally, the folic acid–chitosan coating gave the system the ability to target tumors while preserving healthy tissues.

A pH-sensitive, dual-targeting liposomal system (5-FU-iRGD-FA-pSL) was synthesized by Pandey et al. to increase the effectiveness of 5-FU in the treatment of breast cancer [126]. While the pH-sensitive liposomes allowed for controlled release in the acidic tumor microenvironment, the addition of folic acid and iRGD peptides allowed for tumor-specific targeting and penetration. In MCF-7 cells, the formulation demonstrated improved uptake, cytotoxicity, apoptosis induction, and cell migration inhibition in addition to favorable physicochemical characteristics. Compared to free 5-FU and non-targeted liposomes, in vivo studies further demonstrated enhanced antitumor efficacy, decreased systemic toxicity, and enhanced safety, underscoring its potential as a successful treatment approach for breast cancer [126]. Using glycyrrhizic acid-assisted exfoliation and EDC/NHS coupling, Ha et al. synthesize a folic acid-modified AuNP-MoSe_2_ composite (FAM) for targeted photothermal therapy of triple-negative breast cancer [127]. While the combined action of AuNPs and MoSe_2_ enhanced thermal stability and photothermal conversion efficiency (nearly 62%), folic acid allowed tumor-specific uptake. When compared to unmodified MoSe_2_ composites, in vitro tests using MDA-MB-231 cells showed improved cellular uptake, apoptosis induction under NIR irradiation, and superior therapeutic efficacy, indicating FAM as a promising targeted biomaterial for the treatment of TNBC [127]. The engineering of a folate-gelatin-Poloxamer P407 (FA-Ge-P407) nanogel for the co-administration of paclitaxel and curcumin in breast cancer treatment is described in this study by Ly et al. [128]. FT-IR, HNMR, TEM, and DLS were used to characterize the nanogel, which was created by conjugating FA to Ge-P407. pH-responsive and prolonged drug release was made possible by the encapsulation of paclitaxel and curcumin, with curcumin improving paclitaxel release in acidic environments. When compared to free paclitaxel or single-drug formulations, in vitro tests on MCF-7 cells demonstrated noticeably better cytotoxicity and therapeutic efficacy [128]. These results demonstrate that FA-Ge-P407 nanogels are a promising platform to address drug resistance and paclitaxel limitations in the treatment of breast cancer.

In another study, folic acid-PEG-coated UiO-66 Zr-MOF nanoparticles (UIO-66-EPI@PEG-FA) were prepared by Hashemi et al. to deliver epirubicin (EPI) specifically to breast cancer cells [129]. The system demonstrated a pH-dependent release profile, with increased drug release in acidic tumor-like conditions, and attained a high encapsulation efficiency (approximately 75.3%). By downregulating MMP-2/9 and upregulating caspase-3/9 and mitofusin-1, UIO-66-EPI@PEG-FA activated both apoptotic pathways, induced significantly higher apoptosis, inhibited migration, and altered gene expression in MCF-7 cells. Apoptosis was verified by DAPI staining, and selectivity tests revealed little harm to healthy breast cells. Although more preclinical and clinical testing is necessary, these findings point to UIO-66-EPI@PEG-FA as a promising targeted nanoplatform for breast cancer treatment [129]. Aslam et al. synthesized amphiphilic nanoparticles (FPP for blank and FPPPT for paclitaxel-loaded) using folic acid-grafted poly-3-hydroxybutyrate (FA/PHB), polyethylene glycol, and polyvinyl alcohol [130]. High encapsulation efficiency (approximately 78.8%) and pH-sensitive paclitaxel release were demonstrated by the FPPPT nanoparticles, with increased drug release in acidic environments. In comparison to free paclitaxel, they were biocompatible, showed improved uptake, and significantly increased cytotoxicity, apoptosis, and G2/M cell cycle arrest in MCF-7 cells. These results imply that amphiphilic FPP nanoparticles have potential as tumor-targeted, pH-responsive carriers to improve the effectiveness of paclitaxel in breast cancer treatment [130].

Using a quality by design (QbD) methodology, Rajana et al. reported creating folic acid-decorated, Palbociclib-loaded lipid–polymer hybrid nanoparticles (FA-PLPHNPs) [131]. The optimized formulation showed good stability, high entrapment efficiency (around 93%), and uniform nanosize (143.0 nm). FA-PLPHNPs decreased IC50 values in MCF-7 and MDA-MB-231 cells by 9–11 times when compared to free Palbociclib, confirming increased potency through folate receptor-mediated uptake. Additionally, they inhibited migration and colony formation, increased ROS generation, and induced apoptosis [131]. These results point to FA-PLPHNPs as a promising nanocarrier for targeted treatment of breast cancer. To overcome the pharmacokinetic constraints of pyrimidine analogs, Kunjiappan et al. engineered folic acid-conjugated pyrimidine-2(5H)-thione–loaded curdlan gum-PEGamine nanoparticles (FA-Py-CG-PEGamine nanoparticles) [132]. The crystalline, spherical, 100–400 nm nanoparticles demonstrated a high encapsulation efficiency of 79.0% and pH-dependent release, with a faster rate of drug release at acidic pH (80.0% at pH = 5.4). FA-Py-CG-PEGamine nanoparticles demonstrated potent cytotoxicity (IC50 = 42.3 μg/mL) in MCF-7 cells, improved cellular uptake through endocytosis mediated by the folate receptor, and triggered apoptosis by disrupting mitochondrial membrane potential and producing reactive oxygen species. According to these results, FA-Py-CG-PEGamine NPs show promise as a targeted nanocarrier for the treatment of breast cancer [132].

In a different study, Radhakrishna et al. synthesized folic acid-conjugated, capsaicin-loaded carboxylated multiwalled carbon nanotubes (FA/CAP/COOHMWCNTs) to get around the problems with conventional therapies’ solubility, bioavailability, and targeting [133]. After extensive characterization, the formulation demonstrated a high in silico binding affinity of capsaicin to C-SRC kinase. FA/CAP/COOHMWCNTs showed effective cytotoxicity against MCF-7 cells in vitro (IC50 = 22.7 µg/mL), and in vivo studies in rats with DMBA-induced breast cancer showed marked tumor reduction, increased levels of antioxidant enzymes, and histological restoration of breast tissue. The therapeutic effects of medium-dose treatment were similar to those of doxorubicin, suggesting that FA/CAP/COOHMWCNTs is a promising targeted nanocarrier for the treatment of breast cancer [133]. Nigro et al. developed a mesoporous silica nanodevice (MSN) loaded with bortezomib that releases the drug in acidic conditions and is coated with folic acid to target cancer cells [134]. This system was tested on multiple myeloma cells that overexpress folate receptors and on normal cells lacking these receptors. Unlike free bortezomib (BTZ), the nanodevice did not harm normal cells with limited receptors or alter their metabolism. The control carrier (MSN-FA) was biologically inert in both cell types. Overall, FA-MSN-BTZ demonstrated strong tumor specificity and safety, indicating its promise for targeted multiple myeloma therapy [134].

### 4.4. Folate-Targeted Nanoparticles for Diagnostic Applications

**Figure 7:**
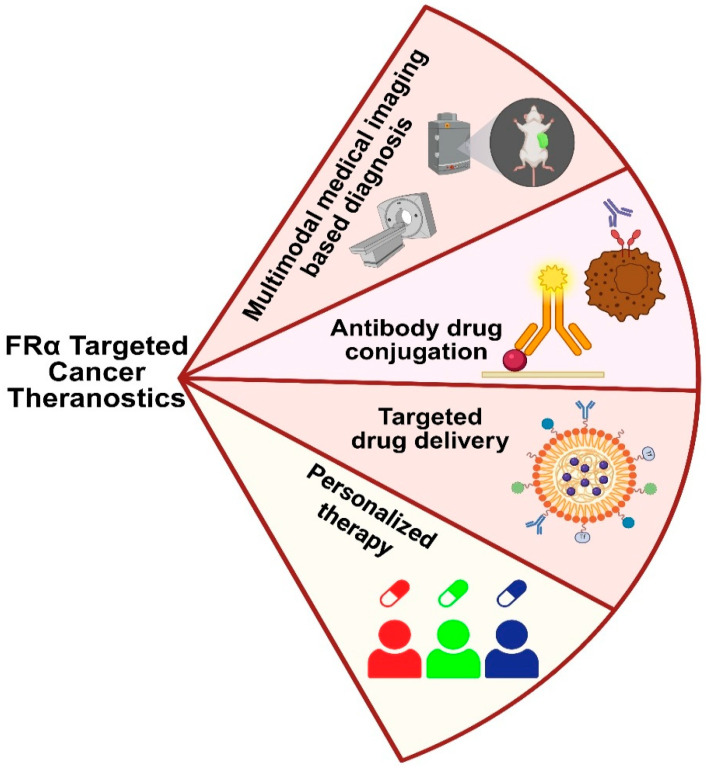
Folic acid receptor-driven theranostic applications in breast cancer that include imaging-based diagnosis, targeted therapy, and personalized treatment.

In addition to evaluating implant integrity, breast imaging is essential for the identification, diagnosis, and clinical treatment of breast cancer [135]. According to recent developments, the primary screening modality for the next decade will continue to be high-resolution, high-contrast x-ray imaging, such as computed tomography (CT), with or without depth information [136]. Ultrasonography and magnetic resonance imaging (MRI) are anticipated to become more important as supplementary procedures, especially for patients with dense breast tissue and high-risk individuals (Figure 7). Although technological constraints still prevent widespread clinical adoption, dedicated breast CT has demonstrated promise in providing high-resolution three-dimensional images [137]. Contrast-enhanced X-ray imaging and nuclear medicine-based methods are becoming useful diagnostic tools for describing suspicious lesions [138]. Furthermore, the potential of optical and electromagnetic modalities to reveal physiological information is being researched, particularly in conjunction with well-established anatomical imaging methods. Phase-sensitive x-ray imaging may improve contrast and edge definition, according to experimental results, but more clinical testing is necessary. Along with a move away from standard screening procedures and toward risk-based, customized imaging approaches, the field is also moving toward multi-modality platforms that incorporate anatomical and functional data. Additionally, new MRI contrast agents, such as manganese and gadolinium-based T1 enhancers and superparamagnetic iron oxide nanoparticles (Fe_3_O_4_) for T2 imaging, have been made easier to create thanks to developments in nanotechnology [139]. Although Fe_3_O_4_ NPs are frequently used in biomedical research, there is still little use for them in the diagnosis of breast and ovarian cancer [140]. Folic acid receptors are overexpressed on many malignant tumors, including breast cancers, and are found in smaller numbers in normal tissues, making targeted imaging techniques using FA ligands particularly intriguing. In breast oncology, this receptor-mediated targeting has a lot of potential for both therapeutic and diagnostic uses [141].

When FA transporters are overexpressed in breast cancer cells, functionalized nanoparticles can deliver contrast agents precisely, improving molecular imaging and early cancer detection. Gadolinium-loaded mesoporous silica nanospheres modified with folic acid (FSG nanoprobes) were fabricated and described in a study by Hosseinabadi et al. [142]. ICP-AES, flow cytometry, and fluorescence microscopy were used to measure their uptake in vitro with different levels of folate receptor expression. The uptake of FSG nanoprobes by cancer cells was approximately 62.0%, which is 2.6 times higher than that of non-targeted nanoprobes. This amounts to 0.6 pg Gd^3+^ per cell without causing cytotoxicity. With a r_1_ relaxivity of 10.1 mM^−1^ s^−1^, the nanoprobes showed great promise for both targeted Gd^3+^ delivery and efficient cancer cell imaging [142]. Upconversion nanoparticles or UCNPs, in particular, are luminescent biolabels that have great promise for bioimaging-based cancer cell detection. Chavez-Garcia et al. synthesized Y_2_O_3_:Yb^3+^,Er^3+^ and Gd_2_O_3_:Yb^3+^,Er^3+^ UCNPs (1%, 10% mol) that can emit red light at 660 nm when excited by 980 nm near-infrared light [143]. To facilitate targeted binding and endocytosis in MCF-7 breast cancer cells that express the folate receptor, the particles were functionalized with aminosilanes and folic acid (UCNP-NH_2_-FA). The findings demonstrated that UCNP-NH_2_-FA was non-toxic and efficiently internalized, with confocal microscopy revealing distinct cytoplasmic localization [143].

In another study, controlled drug delivery systems were developed because drug resistance is still a significant barrier to cancer treatment. Gunduz et al. synthesized magnetic nanoparticles (MNPs) that were loaded with the anticancer medication idarubicin, functionalized with folic acid, and coated with polyethylene glycol [144]. According to light and confocal microscopy, these MNPs showed specific internalization and accumulation in MCF-7 breast cancer cells. Drug-loaded MNPs demonstrated concentration-dependent cytotoxicity and were more effective than free idarubicin, whereas empty MNPs were non-toxic up to 500 μg/mL. The results demonstrate how idarubicin-loaded MNPs may be a useful tactic to improve chemotherapy results [144]. MNPs have the potential to be used in both imaging and drug delivery applications as theranostic agents. Fe_3_O_4_ nanoparticles were synthesized and functionalized with PEG and folic acid in a study by Majd et al. to target cancer cells that express the folate receptor [145]. Tamoxifen (TMX) was then loaded into the nanoparticles. With a sustained TMX release of approximately 90% over 72 h, the resultant Fe_3_O_4_-APS-PEG-FA-TMX nanoparticles (~40 nm) achieved 49.1% drug loading. Their structure was confirmed by characterization, and uptake studies employing flow cytometry and fluorescence microscopy showed that they interacted with MCF-7 breast cancer cells effectively [145]. Significant growth inhibition was demonstrated by cytotoxicity assays, highlighting their potential as multipurpose theranostic agents for targeted imaging and treatment of FR-positive cancers. Cheng et al. used nontoxic CuInS_2_/ZnS quantum dots covalently linked to a Gd(III) complex and functionalized with folic acid for tumor-specific targeting to create a dual-modality magnetic resonance/optical nanoprobe [146]. Aqueous phase transfer was made possible by surface modification using the amphiphilic poly(maleic anhydride-alt-1-octadecene), and Gd(III) conjugation was made easier by carbodiimide chemistry. The nanoprobe produced concentration-dependent MR signal enhancement with a longitudinal relaxivity of 3.7 mM^−1^ s^−1^. After 24 h, cytotoxicity tests revealed that HeLa, HepG2, and MCF-7 cells had more than 80% cell viability at concentrations of up to 100 μg/mL. Effective tumor targeting was demonstrated by confocal imaging, which verified preferential uptake by folate receptor-positive HeLa cells in comparison to HepG2 and MCF-7 [146].

Mortezazadeh et al. coated gadolinium oxide nanoparticles with polyester based on β-cyclodextrin to create a folic acid-targeted MRI contrast agent (Gd_2_O_3_@PCD-FA) [147]. The relaxivity, cytotoxicity, blood compatibility, and tumor-specific imaging of the nanoparticles were all described and assessed by them, which was favorable. Excellent hemocompatibility up to 500 µg Gd^3+^/mL and minimal cytotoxicity against MCF-10A cells up to 50 µg Gd^3+^/mL were confirmed by in vitro tests. It was possible for them to image folate receptor-positive M109 cells using targeted T_1_- and T_2_-weighted MR imaging [147]. In contrast to non-targeted Gd_2_O_3_@PCD, which reached a lower maximum CNR of 1.98 at 6 h and declined to 1.12 by 12 h, in vivo studies revealed that the tumor contrast-to-noise ratio (CNR) peaked at 5.89 within 1 h after Gd_2_O_3_@PCD-FA injection and dropped to 1.45 after 12 h. In a separate study, Zhang et al. fabricated a pH-responsive nanoplatform (Gd-FA-Si) for combined drug delivery and MRI contrast enhancement, utilizing hydroxylated mesoporous silica coated with polyethylenimine, gadolinium, and folic acid [148]. The pH-controlled release of DOX in the simulated bodily fluid was made possible by its loading into the nanopores. A theoretical relaxivity of approximately 1.3 × 10^6^ mM^−1^ s^−1^ was attained by the system due to a high Gd payload (2.6 × 10^5^ per nanoplatform). In comparison to free DOX, folic acid functionalization allowed for targeted uptake in HeLa and MDA-MB-231 cells, which led to more potent cytotoxic effects. These results suggest the potential of the nanoplatform as a pH-sensitive drug carrier and a tumor-specific T_1_ MRI contrast agent for theranostic applications [148].

### 4.5. Folate Receptor-Targeted Immune Therapy

Farletuzumab (MORab003) is a fully humanized monoclonal antibody that is 145 kDa in size and targets the FRα, which is expressed in the ovarian cells of Chinese hamsters as reported by Ledermann et al. [149]. It does not obstruct folic acid binding or FRα-mediated intracellular folate transport, in contrast to many anti-FRα antibodies (Figure 7). Preclinical studies have shown that Farletuzumab induces tumor cell lysis when FRα binds via various pathways, including antibody-dependent cell-mediated cytotoxicity and complement-dependent cytotoxicity. Persistent autophagy and the disruption of FRα–lyn kinase interactions, which inhibit growth signaling pathways, were associated with additional antitumor effects. Patients with platinum-sensitive recurrent ovarian cancer participated in a phase II trial where farletuzumab was evaluated clinically in combination with carboplatin/taxane and then maintenance monotherapy [149]. When used alone, the antibody was well tolerated; when combined with chemotherapy, there was no additional toxicity. Additionally, when compared to previous results from platinum-based doublet chemotherapy, the regimen produced better overall response rates. These advantages were not replicated in later phase III trials in ovarian cancers that were platinum-sensitive or platinum-resistant. The main endpoint of progression-free survival was not met in a sizable phase III trial comparing farletuzumab plus carboplatin/taxane to chemotherapy alone. These results suggest that, although farletuzumab exhibits encouraging biological activity, better patient stratification may be necessary to determine which subgroups are most likely to benefit from FRα-targeted therapy for future success. To enable tumor-selective delivery, cytotoxic drugs are covalently linked to antibodies to create antibody–drug conjugates or ADC. This strategy reduces off-target toxicity by utilizing the antibody’s strong cell-killing action and high binding specificity, which are independent of antibody-dependent cytotoxicity [150]. In contrast to unconjugated antibodies, which are rarely curative on their own, such conjugation improves therapeutic efficacy and permits the clinical use of otherwise too-toxic chemotherapeutics. ADCs’ larger molecular size extends their half-life in the bloodstream, increasing the percentage of the administered dose that reaches and infiltrates the tumor, in contrast to small molecule–drug conjugates, which have short circulation half-lives. An anti-FRα antibody, the cytotoxic payload DM4 (a tubulin polymerization and microtubule assembly inhibitor), and a disulfide linker connecting DM4 to the antibody comprise the first-generation FRα-directed antibody–drug conjugate, IMGN853. After FRα binding, the complex undergoes receptor-mediated endocytosis (RME), in which DM4 is released when the antibody and linker are degraded by lysosomes. Apoptosis and cell-cycle arrest result from the released drug’s disruption of microtubules. For FRα-positive platinum-resistant ovarian cancer, IMGN853 is undergoing phase II clinical evaluation as a monotherapy and in combination therapies. It has demonstrated antitumor efficacy. If clinical results are less than ideal, more chemical optimization may be possible [151].

The human IgG1 antibody m909 was created by Feng et al. and is specific for the FRβ, which is highly expressed on activated macrophages linked to cancer and autoimmune disorders [152]. CHO-hFRβ (FRβ^+^) and CHO-K1 (FRβ^−^) cells were used to confirm binding specificity; flow cytometry revealed selective binding to CHO-hFRβ alone. FRβ selectivity over FRα was confirmed by additional testing on KB nasopharyngeal cells (high FRα expression), which revealed no binding. Antibody-dependent cellular cytotoxicity (ADCC) was demonstrated by functional assays: CHO-hFRβ cells exhibited the highest lysis, preB L1.2 (lower FRβ expression) showed reduced lysis, and CHO-K1 (FRβ^−^) showed none at all when m909 was incubated with NK cells and target lines [152]. However, even at 200 nM, an isotype control IgG was unable to cause cytotoxicity. These results demonstrate that m909 selectively binds cells that express FRβ and causes ADCC by recruiting NK cells. Additionally, CAR-T cells have been used to target FRβ^+^ acute myeloid leukemia (AML) blasts. Lynn et al. developed m909-based CAR T cells for the treatment of acute myeloid leukemia, building on the success of the anti-FRβ antibody m909, as FRβ upregulation is observed in approximately 70% of AML cases [153]. Patient-derived T cells can identify FRβ^+^ tumors with antibody-like specificity thanks to the therapy’s use of the scFv of m909 fused to T cell receptor signaling domains. As demonstrated by in vitro tests, CAR T cells secreted the cytokines IFN-γ, IL-2, TNF-α, and IP-1α when co-cultured with C30-FRβ ovarian cancer cells. ELISA also revealed that when m909 CAR T cells were incubated with FRβ^+^ AML lines, they released more IFN-γ than control CD19-28Z T cells. Using all-trans retinoic acid (ATRA) to increase FRβ expression in AML cells increased cytokine release, suggesting that ATRA treatment and CAR T therapy work in concert. In vivo testing using THPI AML xenograft mice (high FRβ) showed that administering m909 CAR T caused tumor regression [154]. Furthermore, after four weeks, human CD3^+^ T cells demonstrated markedly increased levels in peripheral blood, confirming selective FRβ binding and validating the proliferation of CAR T cells in mice. While research into FR-targeted nanotechnology offers a more comprehensive and flexible platform than antibodies alone, m909 CAR T therapy as a whole shows promise for treating autoimmune disorders and AML.

### 4.6. Folate-Mediated Signaling Pathways

In a seminal study, Boshnjaku et al. examined the novel theory that the folate receptor FRα serves as a transcription factor, in addition to its well-established role as a high-affinity folate transporter [155]. The glycosylphosphatidylinositol (GPI)-anchored protein FRα is primarily found in the plasma membrane’s caveolae fraction. According to their research, when FRα is activated, it translocates from the membrane into the nucleus and directly interacts with cis-regulatory elements in the Fgfr4 (fibroblast growth factor receptor 4) and Hes1 (hairy and enhancer of split 1) promoter regions, thereby altering their transcriptional activity. The FRα recognition domain preferentially binds to AT-rich sequences, indicating sequence-specific DNA interaction, according to detailed promoter mapping. This finding establishes FRα as a dual-function protein with both transport and transcriptional regulatory capabilities, greatly broadening its functional repertoire [155]. The discovery of this nuclear function challenges the conventional understanding of FRα as a folate transporter alone and creates new opportunities to comprehend its role in developmental regulation and possibly in pathophysiological processes like oncogenesis. The tumor-suppressing microRNA miR-34a, often impaired by p53 defects, epigenetic silencing, or gene loss, has strong potential in cancer therapy but faces challenges like instability and toxic, non-specific delivery. Abdelaal et al. developed a fully modified version (FM-miR-34a) that is far more stable and active than unmodified forms [156]. When linked to folate, it precisely targeted tumors, suppressed cancer cell growth and invasion, and even completely cured some mice. This innovation could restore miR-34a’s promise as an effective anti-cancer treatment and justifiy further clinical evaluation. MicroRNA-34a (miR-34a), known for suppressing these stem cells, shows potential as a therapy but faces obstacles like non-specific targeting and toxicity. A promising strategy involves ligand-mediated delivery, such as folate–miR-34a, which has proven effective by targeting FRα [157].

Mohanty et al. found that when FA binds to folate receptor FRα, the specific receptor moves into the nucleus of the cancer cells [158]. There, it works like a transcription factor, switching on Pax3-related genes such as Hes1 and Fgfr4. FRα also boosts the expression of Oct4, Sox2, and Klf4 by attaching to specific regulatory regions in their promoters. Through FRα, FA suppresses certain microRNAs, miR-138 and miR-let-7, which normally inhibit Oct4 and Trim71 (a downstream target of Oct4). This coordinated regulation helps neural crest cells keep their stem-like traits as they migrate during development [158]. Interestingly, this FA-FRα pathway that preserves “stemness” is also active in several cancers, including breast cancer. Disrupting this signaling process could therefore open the door to promising new cancer therapies. DeCarlo et al. demonstrated how the 350 kDa FA-conjugated copolymer (SMA) via a biological linker (DABA) disrupts cellular processes by binding to the folate receptor FRα and lowering the levels of proteins such as p53, STAT3, c-Myc, HES1, and Notch1 [159]. Blocking FRα’s function may indirectly lower STAT3 and c-Myc, which are both associated with drug resistance and cancer cell “stemness,” because it typically aids in the regulation of genes like Oct4. Notch pathways affect the populations of cancer stem cells in breast cancer, and HES1, a target of Notch signaling, also contributes to tumor behavior. As demonstrated in their study of MCF-7 cells, disrupting Notch can reduce these stem-like cell pools, whereas breast cancer stem cells frequently exhibit active Notch signaling. This interference decreased HES1 and NOTCH1 protein levels, caused cell death, changed the structure of triple-negative breast cancer cells (MDA-MB-231), and markedly slowed their motility [159]. The findings demonstrate that the size and shape of the polymer have a significant impact on its capacity to combat cancer. It does this by delivering medications directly to tumor cells and interfering with the molecular pathways that allow them to survive.

**Figure 8:**
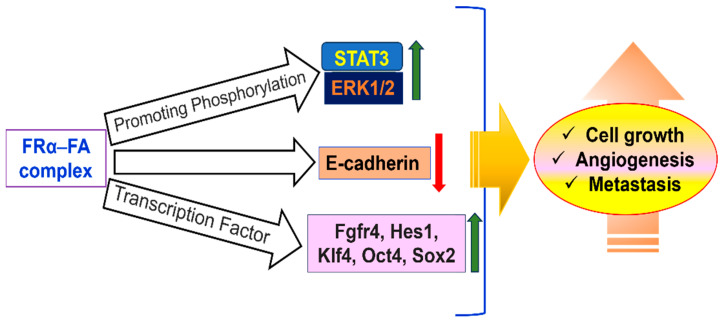
STAT3-activated cell signaling through FRα-FA complex is a key area for targeted cancer therapy.

The folate-modified resveratrol liposomes (FA-Lipo-Res) were developed by Zhu et al. with an average particle size of 118.5 nm and a narrow size distribution [160]. The obtained results showed that adding folate to the liposomes greatly enhanced resveratrol uptake by cancer cells. Therefore, FA-Lipo-Res proved more effective than both free resveratrol and unmodified liposomes (Lipo-Res) in slowing tumor growth, reducing cancer cell migration, and triggering apoptosis. This enhanced effect may be linked to the suppression of the JAK2/STAT3 signaling pathway. In vivo imaging further confirmed that folate modification improved drug accumulation at the tumor site, leading to marked inhibition of tumor growth and metastasis [160]. The proposed pathway is shown in Figure 8 [161].

Although they are restricted by a small number of lysosome-trafficking receptors, lysosome-targeting chimeras (LYTACs) allow targeted protein degradation. Using a polyvalent crosslinking technique, Xiao et al. created FRα-targeting chimeras (FRTACs) after identifying FRα as a novel trafficking receptor [162]. Subnanomolar potency was attained by optimized FRTACs, such as FR-Ctx (EGFR-targeting) and FR-Atz (PD-L1-targeting), whose activity was reliant on FRα expression and lysosomal function. In RM-1 and humanized B16F10 mouse models, FR-Atz reprogrammed the tumor microenvironment toward an immunostimulatory state by promoting T cell–mediated cytotoxicity and inducing strong PD-L1 degradation in vivo, whereas FR-Ctx inhibited the proliferation of cancer cells. These findings establish FRTACs as a potent platform for designing LYTACs that target tumors [162]. A growing number of signaling pathways that control the development of cancer and cellular processes have been linked to folate receptor 1 or FOLR1. Folic acid stimulation causes rapid STAT3 phosphorylation and nuclear translocation in HeLa cells and mouse neural precursor cells, and co-immunoprecipitation confirms FOLR1 interaction with gp130 [163]. This suggests that it plays a role in activating the JAK–STAT pathway. Concurrently, FOLR1 participates in ERK1/2 signaling by interacting with SRC and the progesterone receptor (PGR). This results in the phosphorylation of SRC, ERK1/2, and downstream effectors such as NF-κB, p53, p21, and p27, which regulate cell migration and proliferation in breast cancer cells (MCF-7, T47D) and colon cancer cells (COLO-205) [164]. Furthermore, FOLR1 and TSLC1 work together to mediate MEK/ERK activation in nasopharyngeal carcinoma (HONE1) cells, thereby inhibiting tumor invasiveness. The severity of the disease may also be influenced by FOLR1-driven ERK/FOS-JUN signaling, according to correlative studies in cervical carcinoma tissues. In addition to its role in cytoplasmic signaling, FOLR1 can also function as a transcription factor. After being stimulated by folate, its 38 kDa isoform moves into the nucleus, where it binds to transcriptional regulators like Oct4, Sox2, and Klf4 to increase the expression of pluripotency genes [165]. All of these results demonstrate that FOLR1 is a multifunctional receptor that influences cancer biology and cell fate determination by combining intracellular signaling, transcriptional regulation, and extracellular folate availability.

### 4.7. Folate-Targeted Radioimmunotherapy

Radioimmunotherapy is primarily employed for the treatment of tumors that have metastasized to remote regions of the body; however, it frequently induces significant adverse effects that diminish patient survival rates [166]. Because TNBC has a wide range of molecular and biological behaviors, personalized treatment is still hard to come by, which means that new ways to treat it are needed right away. Recent studies, both preclinical and clinical, indicate that the combination of immunotherapy and radiotherapy may yield a potent synergistic effect. Radiotherapy and immunotherapy can work together to make dendritic cells more active, T-cells more active, and tumor cells more sensitive to radiation by releasing more tumor antigens and presenting them better. Gold nanoparticles (GNPs) have shown a lot of promise as radiosensitizers, but how well they work depends on how well they target tumors. Kefayat and colleagues altered bovine serum albumin-coated gold nanoparticles (BSA-GNPs) with different targeting agents, discovering that glutamine and folic acid-modified variants exhibited superior tumor targeting and radiosensitizing effects in a 4T1 breast tumor mouse model, while maintaining biocompatibility [167]. In addition to targeting drugs, GNPs can be used with other sensitizers to improve the results of radiotherapy even more. For instance, Cheng’s group made gold-titanium dioxide (Au-TiO_2_) nanoparticles that look like dumbbells [168]. These nanoparticles have a high atomic number of gold and the dielectric properties of TiO_2_. This structure makes a lot of ROS, which kills more cancer cells and stops tumors from growing in TNBC models.

Guzik et al. conducted a study utilizing a breast tumor mouse model to evaluate the efficacy of [^177^Lu]Lu-DOTA-folate as an immune stimulator to augment anti-CTLA-4 immunotherapy [169]. The engineered compounds selectively adhered to NF9006 tumor cells and aggregated within tumors. [^177^Lu]Lu-DOTA-folate or anti-CTLA-4 alone did not have much of an effect on tumor growth or survival. But when used together, they slowed the growth of tumors a lot and increased the mice’s average survival time to more than 70 days, compared to 12 days for mice that were not treated. It is important to note that there were no significant side effects. These findings indicate that [^177^Lu]Lu-DOTA-folate may enhance tumor sensitivity to immunotherapy, presenting a promising avenue for the future clinical utilization of folate-based radioconjugates. The study conducted by Smith-Jones et al. evaluated the in vitro and in vivo performance of the radiolabeled monoclonal antibody MORAb-003 as preparation for a clinical trial. Five DOTA molecules were successfully attached to MORAb-003 without affecting its ability to recognize its target. The labeled antibody showed strong binding to the folate receptor alpha on IGROV1 and SW620 cancer cells. Both ^131^I- and ^111^In-labeled MORAb-003 were internalized by these cells; however, while ^111^In remained trapped inside, ^131^I was released as free iodide. In mice, ^111^In-DOTA-MORAb-003 cleared from the bloodstream with a biological half-life of about 110 h, showing strong tumor uptake and similar clearance rates in major organs. A small clinical study with three patients confirmed that ^111^In-MORAb-003 effectively targeted FRα-positive tumors. These findings highlight MORAb-003 as a promising candidate for radioimmunoscintigraphy and radioimmunotherapy of FRα-expressing cancers, alongside its own therapeutic potential.

### 4.8. Challenges to Patient Safety

The progress of FRα-targeted therapies has generated an urgent demand for dependable, reproducible, and quantitative techniques to evaluate folate receptor alpha or FRα expression. This is important because the level of receptor expression has a big effect on how well the treatment works. Nonetheless, various methodological and clinical challenges undermine both the precision of patient selection and overall safety. The main reason for analytical problems is the limits of immunohistochemistry (IHC), which is the most common way to measure FRα in tissue samples [170]. Inconsistent and non-reproducible results can happen because of differences in IHC assay protocols, reagent quality, and imaging platforms and laboratories. Additionally, IHC may not consistently differentiate between functional and nonfunctional receptors, which could result in false-positive outcomes and misguided patient selection for FRα-targeted therapy [171]. This variability makes treatment less precise and could put patients at risk of getting treatments that do not work or are not safe.

From a patient safety and ethical perspective, IHC and other standard molecular assays (including radioligand binding, qPCR, flow cytometry, and electrochemiluminescence) necessitate invasive biopsy techniques. These not only put people at risk of physical harm, like infection, bleeding, or delayed healing, but they also raise moral questions, especially when repeated sampling or metastatic lesions are involved. It is hard to make timely changes to treatment because biopsies cannot be done often enough to check receptor expression [171]. This could make treatment less effective and put patients at risk. In response to these issues, noninvasive imaging techniques utilizing radiolabeled folate analogs have been developed as safer options for detecting and tracking FRα expression. But early imaging agents like ^67^Ga-DF-folate and ^111^In-DTPA-folate had problems with safety and usefulness [172]. ^67^Ga agents showed high levels of accumulation in the intestines, which made it harder to see tumors in the abdomen and made the diagnosis less reliable. ^111^In-based tracers had good contrast, but they were expensive to make and had a long radiochemical half-life (2.81 days), which made it harder for patients to get the tracers and made it harder to use them in a clinical setting.

The advent of ^99m^Tc-based imaging agents, particularly etarfolatide (EC20), has mitigated numerous limitations. ^99m^Tc has good qualities, such as being cheap, having a short half-life (about 6 h), and giving off less energy, which lowers the radiation dose and makes it safer. Etarfolatide only binds to tumors that have FRα, which makes it possible to see the receptor status of the whole body in real time during treatment with its partner drug, vintafolide (EC145) [173]. This integration lowers the need for invasive biopsies and makes patients safer by allowing for dynamic, noninvasive monitoring. However, there are still some problems that need to be solved, such as making sure that imaging sensitivity is the same for all types of tumors, avoiding off-target uptake (especially in kidneys where FRα is normally expressed), and keeping imaging protocols the same across institutions. To protect patients’ safety, it is important to deal with these problems so that they do not get too much radiation, get the right diagnosis, and get the right treatment [174].

## 5. Discussion

Because of its distinct pharmacological and biochemical characteristics, folic acid has become a very promising targeting ligand for tumor-selective drug delivery. Although large drug carriers, such as liposomes or gene vectors, frequently suffer from immunogenicity, rapid clearance by the reticuloendothelial system, delayed extravasation, and predominant passive accumulation in solid tumors, smaller conjugates are better suited to achieve higher tumor-to-normal tissue ratios. This relationship between carrier size and biodistribution and therapeutic efficacy is one of the main challenges in targeted therapy. Folic acid is superior to macromolecular targeting agents like monoclonal antibodies in this regard [175]. Its small molecular size reduces the possibility of immunogenicity and promotes advantageous pharmacokinetic characteristics, enabling repeated administration without unfavorable immune reactions. Folic acid is also inexpensive, easily accessible, and conjugable using straightforward, well-defined chemistries, which makes it a sensible option for large-scale applications. Crucially, the folate receptor is largely absent from most normal tissues and overexpressed on the surface of a variety of human tumors, guaranteeing tumor specificity. Folate also binds to its receptor with high affinity. Furthermore, endocytosis is induced by receptor–ligand binding, facilitating the effective intracellular and possibly subcellular delivery of therapeutic agents [36]. All of these characteristics combine to make folic acid-based targeting systems very appealing for improving the accuracy of cancer treatments and diagnostics, providing a flexible platform for the creation of next-generation nanomedicines.

The folate receptor comes in two main forms—α and β. FRα is often found in higher amounts in epithelial cancers, while FRβ shows up in certain blood cancers and inflamed immune cells. Scientists have found a way to use this to their advantage: by attaching folic acid or anti-FA-receptor antibodies to drug delivery systems, they can specifically target and deliver treatments to FA receptor-positive cancer cells. In breast cancer, FRα is frequently overexpressed, especially in tumors that do not have estrogen (ER) or progesterone receptors (PR). Interestingly, estrogen usually suppresses FRα, which explains why its levels tend to be higher in ER-negative cancers [176]. Drugs like tamoxifen, which block estrogen receptors, can also increase FRα expression. One of the most aggressive breast cancer types, TNBC, lacks ER, PR, and HER2 and makes up 10–15% of cases, but it leads to a high number of deaths due to limited treatment options. Notably, around 80% of TNBC tumors show high FRα levels, making this receptor a promising target for future therapies. It is reported that the effectiveness of folate-targeted therapy in breast cancer varies with the cancer subtype. FRα is highly overexpressed in TNBC and ER- subtypes, making these forms the leading candidates for folate receptor-based treatments [177]. Conversely, FRα expression is relatively low in ER+ breast cancers. To further clarify the role of FRα in breast cancer, Necela et al. analyzed its RNA and protein expression levels across different breast cancer subtypes using next-generation sequencing and immunohistochemistry [178]. The results showed that FRα expression varies widely among subtypes, with certain subsets of each showing elevated levels. Notably, TNBC tumors exhibited significantly higher FRα expression compared to ER+ and HER2+ tumors. Functionally, silencing FRα reduced TNBC cell growth, while its overexpression enhanced folate uptake and promoted proliferation under low-folate conditions. Overall, the findings indicate that TNBC patients with high FRα expression may be ideal candidates for clinical trials involving folate-targeted therapies.

The expression of FRα is highly heterogeneous in breast cancer among different molecular subtypes and patient populations. Elevated FRα levels are characteristic of many triple-negative and ER-negative tumors, and luminal subtypes normally exhibit low to negligible expression, identifying a critical need to stratify patients prior to folate-targeted therapy. That profile of FRα is tumor-associated, but there are also normal polarized epithelial tissues that express FRα with the localization to the apical surface, limiting systemic exposure. However, folate conjugates could be sequestered by non-malignant tissues or bind to FRβ-positive macrophages, allowing for off-target uptake. These limitations, however, can be addressed to a certain degree through rational ligand density optimization, nanocarrier design, and companion diagnostic screening to achieve improved therapeutic precision in FR-targeted drug delivery.

Folate has been attached to a wide range of drug and imaging agent delivery systems like liposomes, lipid and polymeric nanoparticles, micelles, and polymers to help target cancer cells more precisely. Because these carriers are relatively large, a flexible PEG-based linker is often needed to connect the folate and ensure effective targeting. Usually, several folate molecules are attached to each carrier to improve binding strength with folate receptors on breast cancer cells. These systems can stay in the bloodstream longer and avoid being filtered out by the kidneys, but they do face challenges, mainly being cleared by the body’s reticuloendothelial system and struggling to fully penetrate solid tumors. This limits how much drug actually reaches the tumor compared to traditional passive targeting methods that rely on leaky tumor blood vessels, the EPR effect. Additionally, folate-conjugated carriers tend to be cleared faster by the liver. Despite these hurdles, folate-targeted liposomes, lipid nanoparticles, micelles, and even metal-based nanoparticles are opening up new possibilities for more effective breast cancer treatments.

Folate-targeted therapy has a number of benefits that make it a desirable strategy in immunology and oncology. Because the folate receptor, especially FRα and FRβ, is highly overexpressed in many cancers and activated macrophages but has a limited distribution in normal tissues, it can target tumors selectively with little off-target toxicity. Receptor-mediated endocytosis enables dependable intracellular delivery of therapeutic payloads, while its remarkably high binding affinity for folic acid (Kd ~10^−10^ M) guarantees effective drug uptake even at low doses. This approach has demonstrated versatility, supporting platforms like immunotherapies (m909 CAR T cells), nanocarriers (liposomes, nanotubes, bilosomes), small molecule–drug conjugates (like vintafolide), antibody–drug conjugates (like IMGN853), and diagnostic tools (like ^99m^Tc-etarfolatide for SPECT imaging). Crucially, folate targeting is applicable to a variety of tumor types; FRα is common in epithelial cancers, while FRβ is found in tumor-associated macrophages and AML, thereby increasing its usefulness. Additionally, companion imaging agents support precision medicine strategies by facilitating patient stratification and treatment response monitoring. Notwithstanding these advantages, a number of obstacles stand in the way of folate-targeted treatments’ widespread clinical success. Variable or low FR expression is frequently the result of tumor heterogeneity, which restricts drug accumulation and permits the persistence of resistant subpopulations. Long-term therapy may also result in receptor downregulation or alternative uptake pathway activation, which would lessen the therapeutic benefit. When compared to other tumor antigens, the internalization efficiency is limited by the limited number of folate receptors per cell, and high levels of folate in the bloodstream from diet or supplements can interfere with targeting by competing with drug conjugates. Clinical translation is further complicated by the immunogenicity or cytokine release syndrome risks associated with CAR T cells and antibody-based platforms. Lastly, despite being made with selectivity in mind, some off-target toxicity may still occur, especially in normal tissues that are growing quickly or as a result of the linkers’ early drug release. To fully realize the clinical potential of folate-targeted therapy, it will be necessary to optimize receptor selectivity, payload delivery, and patient stratification, even though it is a promising and adaptable modality overall.

Nanomanufacturing is a new and growing field, particularly in nanomedicine, which encompasses small and medium-sized businesses, startups, and large pharmaceutical or medical device companies. Most of these businesses make therapeutic products and systems for delivering drugs. Several nano-enabled drug delivery systems have already been approved, and many more are being tested in clinical trials. However, the number of new nanopharmaceuticals that have been approved has gone down compared to the last ten years [179]. This drop is mostly because it is hard to increase production; current technologies often have trouble making large, consistent, and high-quality batches that are good for clinical testing. To reduce the gap between lab research and clinical use, it is important to set up production that meets Good Manufacturing Practice (GMP) standards. GMP controls not only the manufacturing process but also the whole operational framework, which includes validating equipment, training staff, maintaining hygiene, buying goods, storing goods, following rules, and managing suppliers. GMP is a standardized, reliable framework that is accepted in the EU and US to ensure that quality is maintained throughout the nanomedicine supply chain [179]. It does this by ensuring strict quality control is in place and that risks not identified in final product testing are minimized.

There are many physiological, physicochemical, and molecular factors that affect how toxic nanocarrier systems are. Nanoparticles can enter the body through the skin, lungs, digestive system, or lymphatic pathways. This could lead to cytotoxic or genotoxic effects, but it is not clear how the immune system will respond. These carriers can also change how drugs work by changing their stability, solubility, and pharmacokinetics. As nanotechnology progresses swiftly, regulatory frameworks are still developing to adequately address its environmental, health, and safety (EHS) issues. For safe development and use, we need more thorough toxicological studies, stronger EHS research, and open public discussions about how nanomedicines will affect society as a whole. The nanomedicine market has a lot of promise, but it is still hard to get nanodrugs from the lab to the clinic. Small and medium-sized businesses are responsible for most of the progress, while large pharmaceutical companies are only involved to a small extent. Nanodrugs are expensive because they cost a lot to make, which makes it hard for them to become widely used unless their cost-effectiveness is clearly shown. Even though there is more research being done in this area, there is still no standard way to evaluate cost-effectiveness. This means that new ideas are not being put into practice in the real world. Creating and testing these kinds of models could help make better decisions about policies and cost-effectiveness in the future.

FA-driven breast cancer targeting is still in the early stages of research and testing. There are no folate-functionalized nanocarriers that the FDA or EMA has approved for use in clinical settings. Nehal et al. state that while several in vitro and in vivo studies have shown promising results, such as significantly increased cytotoxicity, higher intracellular uptake, and lower systemic toxicity in folate-modified formulations like FA-PLGA, FA-liposomes, FA-solid lipid nanoparticles, and FA-micelles, there is still no clinical translation because it is hard to reproduce, make on a large scale, and get approval from regulatory bodies [180]. Studies have indicated that FA-targeted formulations of docetaxel, paclitaxel, and doxorubicin resulted in up to 68% tumor growth inhibition and a 1.6- to 2.5-fold enhancement in apoptosis relative to non-targeted systems in breast tumor models. The FDA gave mirvetuximab soravtansine-gynx (Elahere) accelerated approval in 2022. It is the first antibody-drug conjugate that targets FRα. It is made up of an FRα-specific antibody, a cleavable linker, and the cytotoxic agent DM4 (maytansinoid DM4). It is approved for treating adults with FRα-positive, platinum-resistant epithelial ovarian, fallopian tube, or primary peritoneal cancers who have had one to three previous systemic therapies. In general, folate-based targeting strategies, especially those that combine theranostics, photothermal, and photodynamic therapies, are a quickly changing but still preclinical area of breast cancer treatment. They show a lot of promise in experiments, but still need to be tested in real-life situations.

Subsequent studies on folic acid–based delivery of drugs to breast cancer should look at further optimizing the folate-conjugated nanoparticulate vehicles to exploit the overexpression of FR-α in almost 40 to 50% cases of human breast tumors. Indeed, pH- or redox-sensitive folate-modified liposomes, polymeric nanoparticles, or lipid–polymer hybrids enable enhanced receptor-mediated uptake, increased drug loading efficiency, and controlled intracellular release. Integrating co-delivery formulations that deliver chemotherapeutics and siRNA will be an added advantage in combating resistance mechanisms. Also, a comparative summary of nanocarrier features, i.e., particle size, surface charge density or zeta potential, loading efficiency, release kinetics profiles, and cellular uptake data would benefit the faster growth of targeted delivery systems for the treatment of breast cancer, where RF-receptor might play a major role. Essentially, it is a future proposal to establish a library focusing on the physicochemical properties of nanoformulations or engineered nanoparticles for FA-targeted drug delivery for breast cancer therapy, with the potential to inform researchers on how to fabricate and easily translate these findings into clinical practice.

## 6. Conclusions

Traditional chemotherapy works by attacking fast-dividing tumor cells, but it often harms healthy tissues too, leading to serious side effects. These treatments also face challenges like drug resistance and a narrow margin between effective and harmful doses. In contrast, targeted therapies, as well as diagnosis, offer a smarter approach. They can identify cancer cells with specific receptors, like the folate receptor (FR), and help tailor treatments to patients who are most likely to benefit. Folic acid receptors, especially the FRα type, are highly overexpressed in several solid tumors, including breast cancer, and tend to increase as the disease progresses. High levels of FRα are also linked to better outcomes in breast cancer therapies. This makes folic acid receptors a promising target for more precise, personalized cancer treatments as well as diagnosis using folic acid-linked nano drug delivery vehicles that specifically home in on FR-positive breast tumors.

## Figures and Tables

**Table 2:** Nanoformulated chemotherapeutics for the treatment of breast cancer.

Nanostructures/Modifications	Chemotherapeutics/Payloads	Potential Applications	Limitations	Ref.
Janus nanostructure composed of liposomes and porous and amorphous zeolitic imidazole framework (AZIF-8)	AZIF-8 encapsulated with Dox & Liposomes with mitoxantrone coated with cell membrane & aptamer	Dual-targeted breast cancer therapy with the least side effects, tumor suppression in BALB/c mice	Complex synthesis and safety concern	[68]
Biomimetic nanocomplex combining cationic liposomes & macrophage-evolved exosomes	Docetaxel & Bcl-2 siRNA	ROS-responsive co-delivery induces apoptosis in tumor cells, increasing circulation half-life.	Long-term toxicity and immune response	[69]
Biodegradable chitosan nanoparticles	Tetraphenylchlorin conjugation, combining ferroptosis inducer RSL3	Boosts cellular stress & respiration, with mesenchymal-like breast cancer cells showing high ferroptosis susceptibility	In vivo validation and safety issues	[70]
Self-assembled polymer made of amino-functionalized hydroxyethyl starch grafted with cholesterol.	Flucoxanthin & siRNA	Remodeled TME, synergistically killed tumor cells, reduced TNBC tumor burden, and inhibited lung metastasis.	Biocompatibility and clinical validation	[71]
PEGylated Mn-coordinated nanoparticles	Chlorin e6 and STING agonists MSA-2	Phototherapeutic effect, induced immunogenic cell death, overcoming antitumor immunity.	Concentration-dependent manganese toxicity	[72]
FCPCV nanoparticles & CRISPR-Cas9	sgRNA targeting CCL5	Silenced CCL5, boosted CD8+ T cell activity, improved cytokine production, suppressed tumor, promising personalized breast cancer immunotherapy.	Off-target gene editing	[73]
Tetrahedral DNA nanoparticles	siOTUD6B and Dox	Controlled release & improved cellular uptake of Dox, inhibits metastasis	In vivo stability of DNA-based nanostructures	[74]
Human serum albumin (HAS)-α-tocopherol succinate nanoparticles	Lapatinib & letrozole	Inhibit tumor development and induce apoptosis.	Long-term safety	[75]
Chitosan nanoparticles	5-FU and trastuzumab	Targeting SK-BR-3 cell line, 85.2% tumor inhibition, efficiently targeted breast cancer therapeutics.	Clinical validation and immunogenicity	[76]
Camouflaged liposomes functionalized with iRGDP.	Trastuzumab, Gefitinib, lycorine hydrochloride	Improved IC50 against MCF-7 cells, a potential multifaceted therapy against metastatic breast cancer.	Comprehensive pharmacokinetics and toxicity	[77]
Platelet membrane encapsulated biomimetic iron oxide nanoparticles	Paclitaxel	pH-responsive drug delivery, targeted magnetic hyperthermia, IC50 values reduced to 1 μg/mL & more than 92% tumor growth inhibition	In vivo safety and toxicity	[78]
Iron oxide nanoparticles	Curcumin	Photodynamic therapy mediated tumor cell death, with 32% cell viability at 30 mg/mL concentration.	Detailed mechanistic assessment	[79]
pH-responsive magnetic-chitosan core–shell nanoparticles	Methotrexate	pH-responsive drug release significantly enhanced the anticancer effect.	In vivo toxicity validation	[80]

## Data Availability

No new data were created or analyzed in this study.
